# Design, Synthesis and Actual Applications of the Polymers Containing Acidic P–OH Fragments: Part 2—Sidechain Phosphorus-Containing Polyacids

**DOI:** 10.3390/ijms24021613

**Published:** 2023-01-13

**Authors:** Ilya E. Nifant’ev, Pavel V. Ivchenko

**Affiliations:** A.V. Topchiev Institute of Petrochemical Synthesis RAS, 29 Leninsky Pr., 119991 Moscow, Russia

**Keywords:** biocompatibility, coordination polymerization, free-radical polymerization, metathesis polymerization, polycondensation, polymer composites, phosphinic acids, phosphoric acids

## Abstract

Macromolecules containing acidic fragments in side-groups—polyacids—occupy a special place among synthetic polymers. Properties and applications of polyacids are directly related to the chemical structure of macromolecules: the nature of the acidic groups, polymer backbone, and spacers between the main chain and acidic groups. The chemical nature of the phosphorus results in the diversity of acidic >P(O)OH fragments in sidechain phosphorus-containing polyacids (PCPAs) that can be derivatives of phosphoric or phosphinic acids. Sidechain PCPAs have many similarities with other polyacids. However, due to the relatively high acidity of –P(O)(OH)_2_ fragment, bone and mineral affinity, and biocompatibility, sidechain PCPAs have immense potential for diverse applications. Synthetic approaches to sidechain PCPAs also have their own specifics. All these issues are discussed in the present review.

## 1. Introduction 

Polymers containing multiple acidic (–C(O)OH, S(O)_2_OH, –P(O)(OH)_2_, etc.) fragments distributed throughout the polymer backbone—polyacids, more commonly named anionic polyelectrolytes—constantly attract the researchers’ attention [1,2,3]. Enhanced hydrophilicity, proton conductivity, ability to complexation with metal ions and organic bases, and biocompatibility are occasionally shown by this type of polymer—this is not a complete list of attractive properties of polyacids that determine a variety of their applications. Phosphorus-containing polyacids (PCPAs) represent a separate group of polyanionic electrolytes due to the relatively high acidity of the –P(O)(OH)_2_ group, higher biocompatibility, and bone and mineral affinity of phosphates. PCPAs can be classified into main-chain PCPAs (polyphosphodiesters, discussed in Part 1 of the review) and sidechain PCPAs that represent macromolecules containing acidic phosphate or phosphonate fragments as substituents distributed throughout the polymer backbone. Sidechain PCPAs have more ‘degrees of freedom’ in polymer designs when compared with polyphosphodiesters; however, not all possible synthetic approaches to sidechain PCPAs have been realized to date.

Some issues related to chemistry [4,5,6,7,8,9,10] and applications [6,11,12,13,14] of the sidechain PCPAs have been addressed fragmentary in previously published reviews. In the present work, we tried to cover the issues of the synthesis and actual applications of sidechain PCPAs more exhaustively.

## 2. Design and Synthesis of Sidechain PCPAs

### 2.1. Synthetic Approaches to Sidechain PCPAs: An Overview

**Scheme 1:**
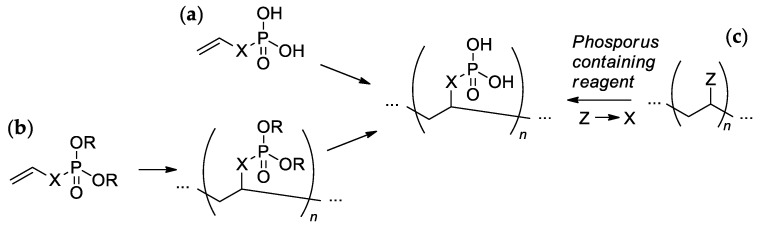
The common synthetic approaches to sidechain PCPAs: (**a**) polymerization of unsaturated phosphoric or phosphinic acids; (**b**) polymerization of unsaturated phosphoesters followed by hydrolysis; (**c**) phosphorylation of polymers.

Most of the synthetic approaches to sidechain PCPAs use (co)polymerization of the phosphorus-containing vinyl monomers (Scheme 1a,b). The alternative approach is based on the post-modification of the (co)polymers (Scheme 1c). These main approaches are reviewed and discussed in more detail in Section 2.3 and Section 2.4, respectively. Several other specific methods (e.g., acyclic diene metathesis (ADMET) polycondensation and ring-opening metathesis polymerization (ROMP)) that found their application in particular cases are discussed in Section 2.5. 

### 2.2. Phosphorus Containing Vinyl Monomers

Vinyl monomers, containing phosphonate or phosphate fragments, are definitely starting compounds suitable for the synthesis of sidechain PCPAs, and that was the reason why we chose to devote a separate section to these compounds. The synthesis and chemistry of the phosphorus-containing vinyl monomers were discussed previously in the review of Macarie and Ilia [7], outlining poly(vinylphosphonic acid) and its derivatives, and in the review of David and Coll. [10], devoted to phosphonate vinyl monomers and polymers. Monomers and PCPAs containing fluoro substituents in the main polymer chain were mentioned in the review of Améduri and coll. [9]. A representative list of vinyl monomers suitable for the preparation of adhesive (co)polymers for dentistry was presented in the work of Moszner, Salz, and Zimmermann [5].

#### 2.2.1. Vinylphosphonic Acid and Related Compounds

**Scheme 2:**
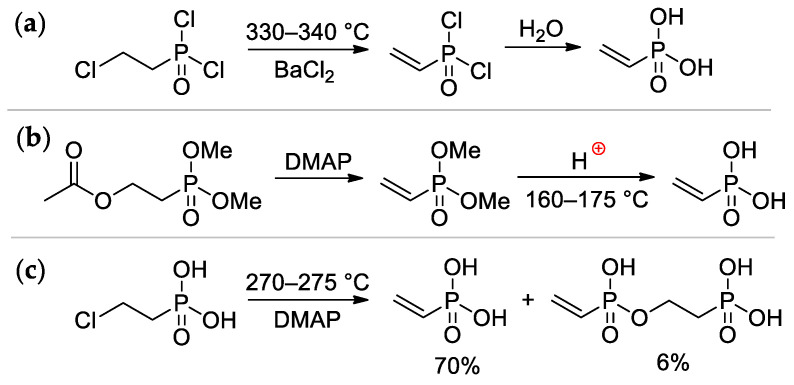
The synthesis of vinylphosphonic acid: (**a**) from (2-chloroethyl)dichlorophopshonate [15]; (**b**) from AcOCH_2_CH_2_P(O)(OMe)_2_ [17]; (**c**) based on commercially available(2-chloroethyl) phopshonic acid [18].

Vinylphosphonic acid (VPA) CH_2_=CHP(O)(OH)_2_ represents the simplest monomer for the synthesis of sidechain PCPAs [7]. It was obtained for the first time by Kabachnik and Medved in 1959 via dehydrochlorination of ClCH_2_CH_2_P(O)Cl_2_ (330–340 °C, BaCl_2_) followed by hydrolysis of CH_2_=CHP(O)Cl_2_ intermediate [15] (Scheme 2a); in turn, ClCH_2_CH_2_P(O)Cl_2_ was prepared by the reaction of PCl_3_ with oxirane, rearrangement of the resulting P(OCH_2_CH_2_Cl)_3_ to ClCH_2_CH_2_P(O)(OCH_2_CH_2_Cl)_2_, followed by its reaction with thionyl chloride [16]. Another method was based on the reaction of AcOCH_2_CH_2_P(O)(OMe)_2_ with DMAP, followed by acidic hydrolysis of the CH_2_=CHP(O)(OMe)_2_ intermediate [17]. (Scheme 2b). The cost-effective synthetic approach was based on a readily available starting compound ClCH_2_CH_2_P(O)(OH)_2_ (used as a growth regulator for plants), its pyrolysis was accompanied by the formation of the bis(phosphonate) side product [18] (Scheme 2c). The use of microwave irradiation in the pyrolysis of ClCH_2_CH_2_P(O)(OH)_2_ proved ineffective [19]. Vinylphosphonic acid is a colorless, low-melting solid (M.p. 36 °C) [20].

**Scheme 3:**
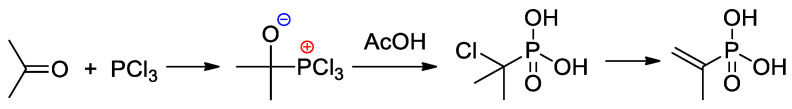
The synthesis of 2-propenylphosphonic acid [21].

An efficient method of the synthesis of 2-propenylphosphonic acid involves the nucleophilic addition of PCl_3_ to acetone followed by the reaction with AcOH and subsequent dehydrochlorination [21] (Scheme 3).

#### 2.2.2. Phosphorylated Acrylates and Related Compounds

The high reactivity of the esters of acrylic and methacrylic acids in polymerization and their greater synthetic availability in comparison with VPA and VPA esters are the reasons for researchers’ continued interest in vinyl monomers of this type. Among others, derivatives of (2-hydroxyethyl)methacrylate (HEMA) deserve special mention due to their synthetic availability.

**Scheme 4:**
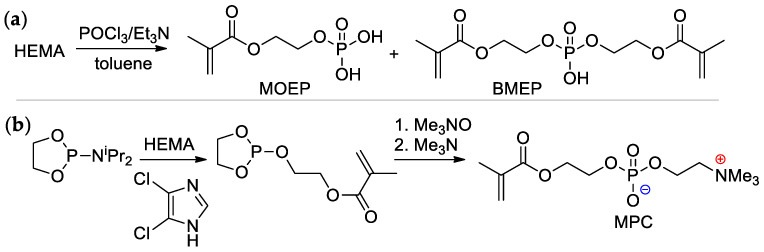
Synthesis of (meth)acrylates containing –P(O)(OH)_2_ group in alkoxy fragment: (**a**) phosphoryl chloride based approach; (**b**) 1,3,2-dioxaphospholan-2-amine based approach [22,23,24].

**Scheme 5:**
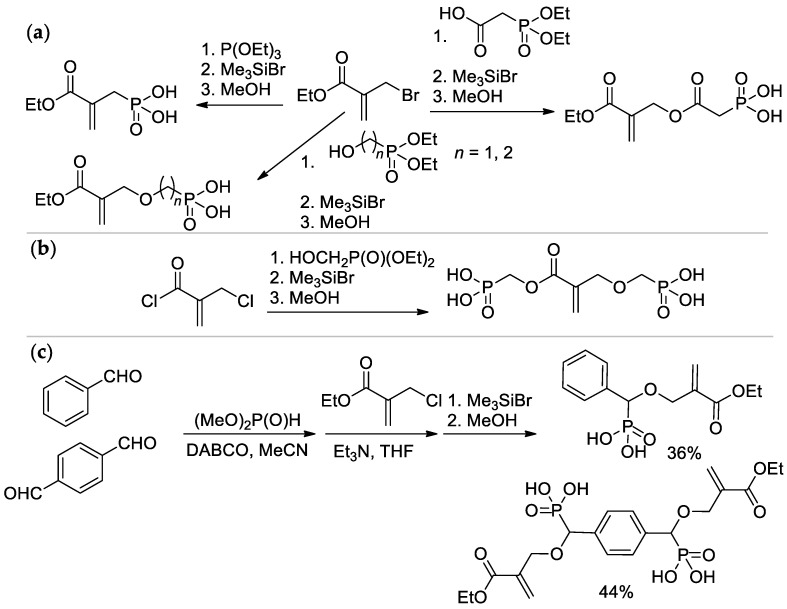
Synthesis of phosphonated methacrylates via functionalization of the methyl group: (**a**) based on ethyl 2-(bromomethyl)acrylate; (**b**) from 2-(chloromethyl)acryloyl chloride; (**c**) via condensation of aldehydes with dimethyl phosphonate.

Different synthetic approaches to phosphate- and phosphonate-functionalized acrylates are presented and discussed below. Evidently, phosphate or phosphonate groups can be introduced as a part of the alkoxy fragment of (meth)acrylate (the easiest and most affordable way, Scheme 4), but functionalization of the methyl group of methacrylate also leads to prospective monomers (Scheme 5).

2-(Methacryloyloxy)ethyl phosphate (MOEP) and bis[2-(methacryloyloxy)ethyl]phosphate (BMEP) were synthesized by the reaction of HEMA with POCl_3_, followed by hydrolysis, the yields of the products depended on the HEMA/POCl_3_ ratio [22,23] (Scheme 4a). Direct condensation of HEMA with phosphorylcholine is accompanied by polymerization, and the synthesis of methacryloyl phosphorylcholine (MPC) was carried out using the ‘phosphoramidite’ route with an overall yield of 37% [24] (Scheme 4b).

The Michaelis–Arbuzov reaction of α-bromo ethyl methacrylate with P(OEt)_3_ with subsequent hydrolysis resulted in the formation of (2-(ethoxycarbonyl)allyl)phosphonic acid [22,25], while its acylation by 2-(diethoxyphosphoryl)acetic acid and hydrolysis allowed to obtain (2-((2-(ethoxycarbonyl)allyl)oxy)-2-oxoethyl)phosphonic acid [26]; alkylation and hydrolysis lead to ether-linked acrylic phosphonates [27] (Scheme 5a). Interaction of 2-(chloromethyl)acryloyl chloride with hydroxymethyl diethyl phosphonate afforded bis(phosphinic acid) [25] (Scheme 5b). Synthesis of aromatic acrylic phosphonates was carried out by the Pudovik reaction, starting from benzaldehyde or terephthaldialdehyde [22,27] (Scheme 5c).

**Scheme 6:**
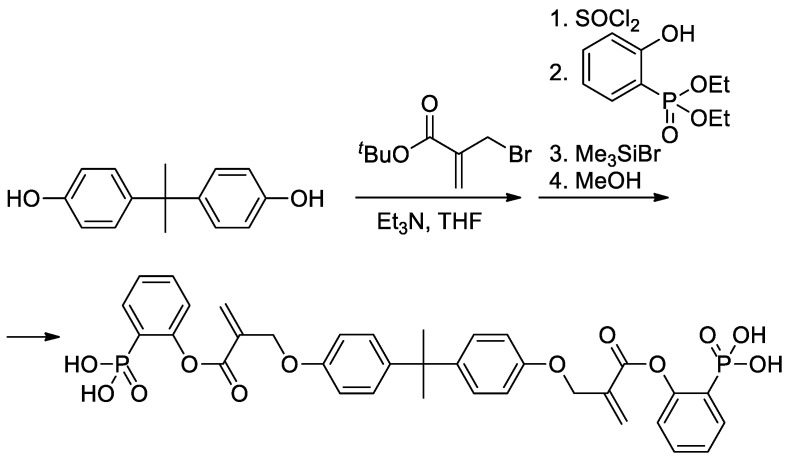
Synthesis of bis(phosphonic acid) from Bisphenol A [28].

As can be seen in Scheme 5b, both alkoxy and methyl groups of methacrylate can be affected by modification. The applicability of this approach was also demonstrated by Avci and coll et al., who used the example of the synthesis of Bisphenol A-based bis(phosphonic acid) (Scheme 6) [28].

**Scheme 7:**
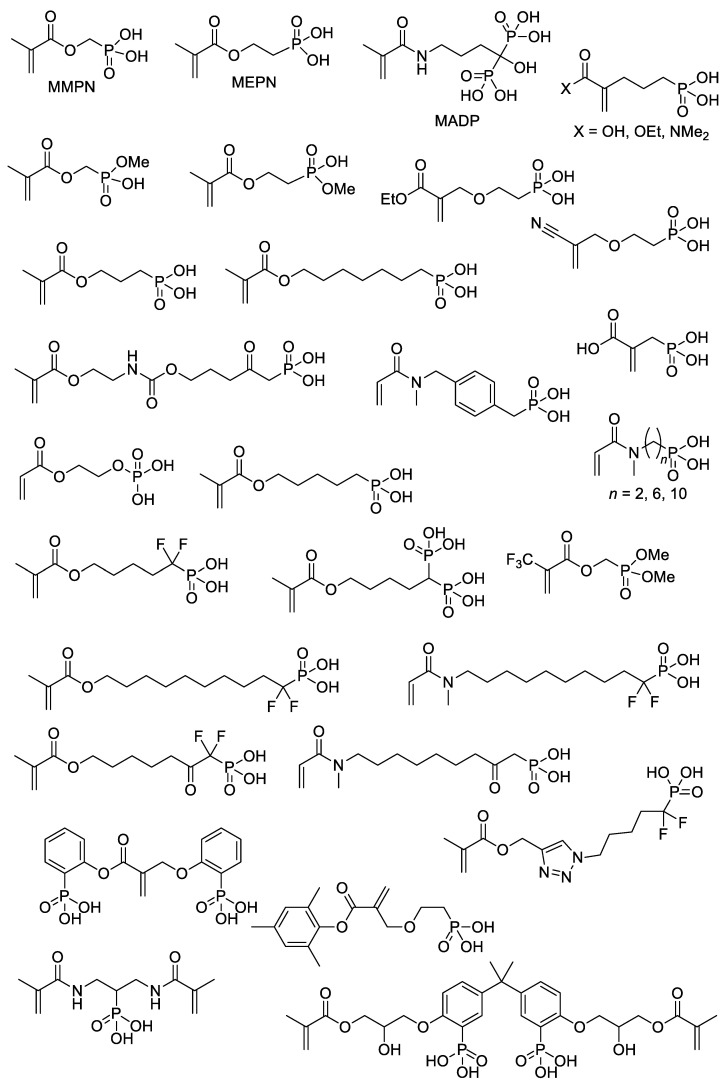
The structures of the (meth)acrylates containing –P(O)(OH)_2_ fragments [22,29,30,31,32,33,34,35,36,37,38,39,40,41,42,43].

With the use of common synthetic approaches (Scheme 4 and Scheme 5) and other equally simple reactions, dozens of –P(O)(OH)_2_-functionalized acrylates were synthesized [22,29,30,31,32,33,34,35,36,37,38,39,40,41,42,43] (Scheme 7). An important note is that an incomplete list of acrylates, presented in Scheme 7, can be further expanded by introducing dialkyl phosphonate since deprotection with the formation of PCPAs can be easily carried out after polymerization [44,45]. However, a number of acrylate monomers containing –P(O)(OR)_2_ groups and polymers obtained on their basis were not studied in the synthesis of PCPAs [46,47].

#### 2.2.3. Other Phosphorus Containing Monomers

Fluorinated polymers represent a separate group of materials due to specific surface characteristics and high thermal and chemical stability. In a recent review by Améduri et al. [9], different synthetic strategies for fluorinated (co)polymers containing phosphorus groups are discussed, with the consideration of –P(O)(OH)_2_-functionalized monomers and polymers. However, the synthesis of such type compounds was described in a single article by Yamabe et al. who developed a complex and time-consuming method for the synthesis of perfuorovinyloxy-substituted perfuoroalkylphosphonic acid derivatives CF_2_=CFO(CF_2_)_3_P(O)(OMe)_2_ [48].

**Scheme 8:**
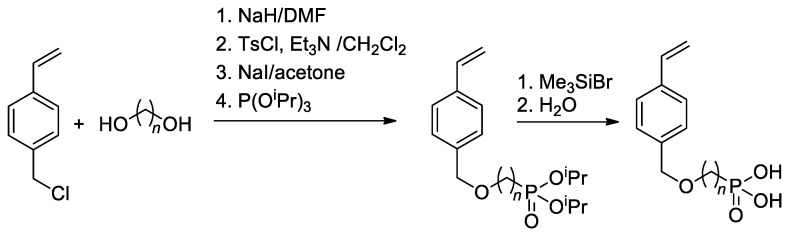
Synthesis of styrene-derivative monomers containing the phosphonic acid [49].

Since substituted styrenes are highly active in all types of polymerizations, the idea of using –P(O)(OH)_2_-functionalized styrenes in polymerization is certainly feasible. Based on 4-(chloromethyl)-styrene, Yoon and Coll. [49] have synthesized styryl phosphonic acids with different lengths of the alkanediyl spacers between the benzene ring and –P(O)(OH)_2_ group (Scheme 8). To introduce CH_2_CF_2_ linkage between styrene and phosphonate fragments, Alter and Hoge used the reaction of 4-(iodomethyl)styrene with LiCF_2_P(O)(OEt)_2_ [50].

**Scheme 9:**
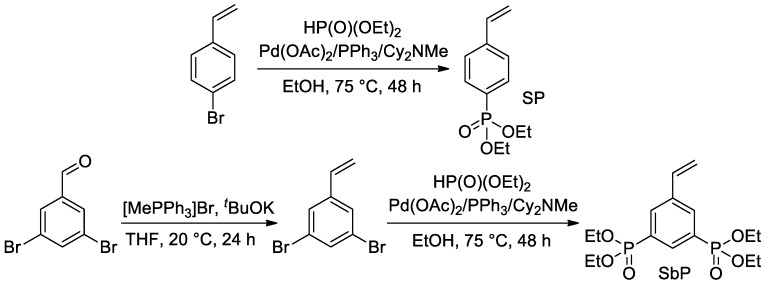
Synthesis of styrenes with one or two –P(O)(OEt)_2_ substituents [51].

Later, Park et al. proposed the use of Pd-catalyzed cross-coupling for the direct introduction of one or two –P(O)(OEt)_2_ fragments into an aromatic ring with a formation of styryl phosphonate and bis(phosphonate) (SP and SbP, respectively) [51] (Scheme 9). Hydrolytic cleavage of the diethyl phosphonate fragments was planned and realized after polymerization (see Section 2.3.4).

**Scheme 10:**
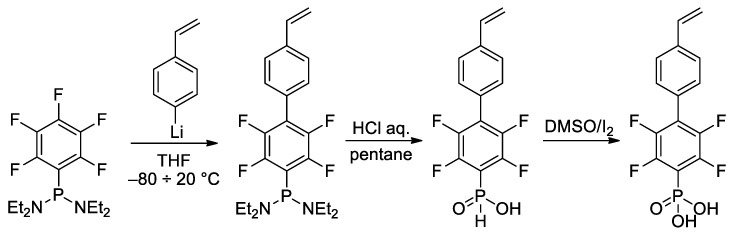
Synthesis of phosphonic acid with 1,4-perfluorophenylene linkage between styryl and –P(O)(OH)_2_ fragments [52].

**Figure 1:**
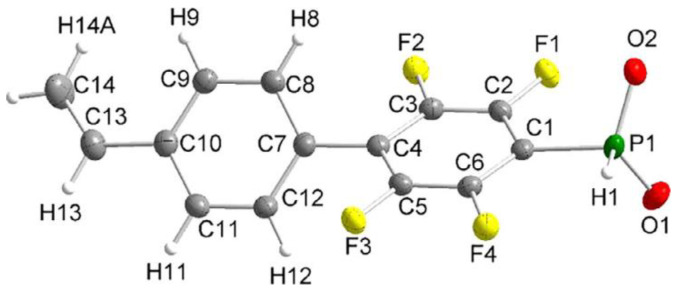
Molecular structure of the anion of the diethyl ammonium salt of phosphinic acid with 1,4-perfluorophenylene linkage between styryl and –PO_2_H^−^ fragments (thermal ellipsoids are set to 50% probability; cation omitted.). Reprinted with permission from [52]. Copyright (2020) Wiley-VCH GmbH.

In the study of Hoge et al. [52], the idea of the use of styrene-derived phosphonates was further developed. Considering the high *para*-selectivity of nucleophilic substitution in N,N,N′,N′-tetraethyl-1-(perfluorophenyl)phosphinediamine, they synthesized vinyl monomer having 1,4-perfluorophenylene linkage between styryl and –P(O)(OH)_2_ fragments (Scheme 10). Note that intermediate phosphinic acid is virtually the only phosphorus-containing ‘monomer’ with a proven molecular structure (Figure 1).

Dialkyl(alkenyl)phosphates, readily available by the reaction of enolates with ClP(O)(OR)_2_ [53,54], can also be considered as monomers for the synthesis of sidechain PCPAs; however, their formation is complicated by the rearrangement to β-ketophosphonates [55,56].

### 2.3. Homopolymerization and Copolymerization of the Phosphorus Containing Vinyl Monomers

#### 2.3.1. Homopolymerization of VPA, Its Derivatives and Analogs

Since poly(vinylphosphonic acid) (PVPA) continues to attract the researchers’ attention based on new fields of PVPA can be synthesized via radical polymerization of vinylphosphonic acid in the presence of initiators and optionally chain transfer agents (CTAs) with different functionality and reactivity.

**Scheme 11:**
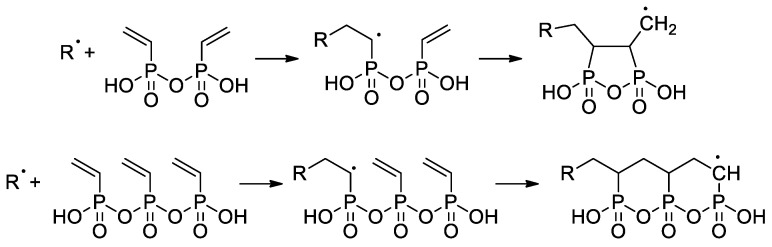
Formation of the side products during polymerization of VPA resulting from intermediate formation of the VPA anhydrides [57,59].

Wegner et al. [57] conducted a series of experiments on VPA polymerization in aqueous media using α,α′-azodiisobutyramidine dihydrochloride (AIBA) as an initiator. In contrast to free-radical polymerization of other polar vinyl monomers, polymerization of VPA occurred with low regioselectivity, and a probability of head-to-head and tail-to-tail links over regular head-to-tail links of 23.5% was obtained. When using AIBN as an initiator and ethyl acetate as a solvent, PVPA containing up to 17% head-to-head fragments was obtained [58]. To explain the low regioselectivity of VPA polymerization, Wegner et al. [57] suggested that the process may occur according to two different mechanisms, i.e., a classical head-to-tail radical polymerization and a cyclopolymerization of the VPA anhydride formed in situ (Scheme 11). Further studies on VPA polymerization in Ac_2_O media, conducted by Voit and Coll. [59], have shown that the intermediate formation of anhydride results in an acceleration of the reaction due to higher reactivity of the VPA anhydride with a formation of cyclic side-products (Scheme 11). The formation of anhydride fragments (up to 19 wt%) was also detected when the polymerization of VPA was conducted in ethyl acetate with the use of a benzoyl peroxide initiator [60]. AIBA-initiated homopolymerization of VPA was used in the preparation of PVPA samples suitable for composition with different inorganic compounds [61,62,63].

The use CTAs in the radical polymerization of VPA was reported for the first time by David and Coll. [64]. Only CBrCl_3_ successfully served as CTA in the synthesis of telechelic VPA oligomers with yields of ~70% and *DP*_n_ = 6–60. High-MW PVPA (*M*_n_ 42.2 kDa) was obtained in aqueous media at 80 °C when using a 620:1 VPA/AIBA ratio [65].

Prospective results were received when using *n*-C_6_F_13_I as a CTA that exhibited CF_2_I functionalization of the oligomer obtained. MALDI-TOF MS and NMR analysis indicated that conventional radical initiation and termination do not occur and that the oligomers were obtained from transfer reactions [66].

**Scheme 12:**
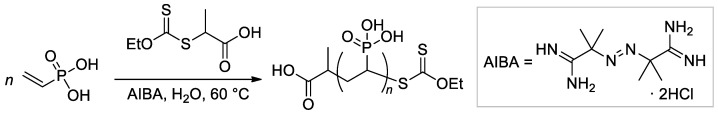
Aqueous RAFT/MADIX polymerisation of VPA mediated by a carboxy-functional xanthate [67].

The applicability of reversible addition-fragmentation chain transfer/macromolecular design via the interchange of xanthate (RAFT/MADIX) approach to radical polymerization of VPA was studied by Destarac and Coll. [67]. They demonstrated that at 65 °C in water carboxy functionalized O-ethyl xanthate reacts rapidly and quantitatively with VPA oligoradicals to yield low-MW O-ethyl xanthate-capped PVPAs (Scheme 12), and later, reversible transfer of the xanthate terminal group leads to an increase of *M*_n_ during polymerization. AIBA was used as a water-soluble initiator of radical polymerization. A follow-up study by the same group has shown that in the presence of 0.25-0.75, molar equivalents of NaOH, the rate and the final conversion of both conventional and RAFT polymerizations were increased, and the fastest rates of polymerization were obtained in the presence of 0.5 equivalents of NaOH [68].

The other possibility to obtain PVPA consists in the polymerization of vinylphosphonic acid derivatives CH_2_=CHP(O)Cl_2_ or CH_2_=CHP(O)(OR)_2_ followed by hydrolysis. As was demonstrated by Ellis and Wilson, PVPA can also be obtained by free-radical polymerization of CH_2_=CHP(O)Cl_2_ with the use of an AIBN initiator, followed by hydrolysis [69]. However, to provide acceptable mechanical characteristics of PVPA-based materials, the relatively low-MW polymer thus obtained was forcedly subjected to the treatment with cross-linking reagents [70].

**Scheme 13:**
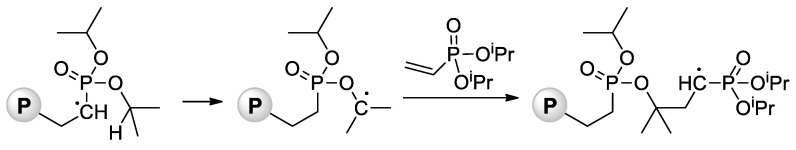
Intramolecular hydrogen transfer from the backbone to the side chain with formation of P–O–C bonds during radical polymerization of diisopropyl vinyl phosphonate (P = polymer) [72].

Jin and Gonsalves reported a relatively low *M*_n_ value of 8 kDa for homopolymer formed during AIBN-initiated bulk polymerization of CH_2_=CHP(O)(OMe)_2_ [71], the microstructure of the polymer was not determined. During the study of AIBA-initiated polymerization of CH_2_=CHP(O)(OMe)_2_, Wegner et al. [57] showed higher regioselectivity of this reaction in comparison with VPA homopolymerization. For the transformation of the polymer obtained into PVPA, it reacted with an excess of aq. HBr at 110 °C for 8 h. Note that radical polymerization of CH_2_=CHP(O)(O^i^Pr)_2_ can be complicated by intramolecular hydrogen transfer from the backbone to the side chain [72] (Scheme 13).

The study of UV-induced copolymerization of VPA with CH_2_=CHP(O)(OMe)_2_ [73] deserves to be mentioned: in the presence of 3 wt% Darocur 4265 photoinitiator (that represents a mixture of (diphenylphosphoryl)(mesityl)methanone and 2-hydroxy-2-methyl-1-phenylpropan-1-one), VPA or VPA/phosphonate mixtures were laid on PTFE plates and exposed to UV irradiation. PVPA with *M*_n_ = 27.75 kDa (*Ð*_M_ = 1.23) was obtained; for copolymers, *M*_n_ values increased from 8.78 to 16.12 kDa with an increase in the VPA/CH_2_=CHP(O)(OMe)_2_ ratio from 1:1 to 4:1. ATRP was also used in the synthesis of core-shell materials with the use of oligo-(CMe_2_Br) substituted polyaromatics and CH_2_=CHP(O)(OEt)_2_ [74].

**Scheme 14:**
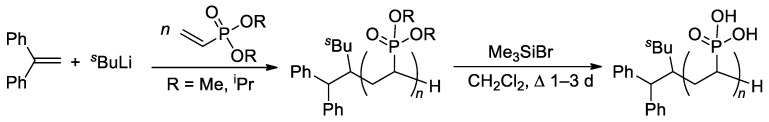
Synthesis of PVPA by anionic polymerization of dialkyl vinyl phosphonate, followed by the cleavage using Me_3_SiBr [75].

The relatively low reactivity of CH_2_=CHP(O)(OR)_2_ in free-radical polymerization has heavily conditioned the search for alternative methods of polymerization of dialkyl vinyl phosphonates. Meyer and Coll. proposed the use of *^s^*BuLi and Ph_2_C=CH_2_ as an initiator of the anionic polymerization of CH_2_=CHP(O)(OR)_2_ (R = Me, ^i^Pr) in THF [75]. High yields of the polymers were obtained when using diisopropyl vinyl phosphonate, while CH_2_=CHP(O)(OMe)_2_ gave only low yields due to the precipitation of the polymer already in low conversions. Anionic polymerization proceeded regioselectively, but with low stereoselectivity. Given that mild and quantitative hydrolysis of dialkyl phosphonates can be performed via the intermediate formation of silyl esters [76], polyesters were refluxed with Me_3_SiBr in CH_2_Cl_2_ to yield PVPA samples of different *M*_w_ (4.2–814 kDa) and *Đ*_M_ of 2.1–3.9 (Scheme 14).

**Figure 2:**
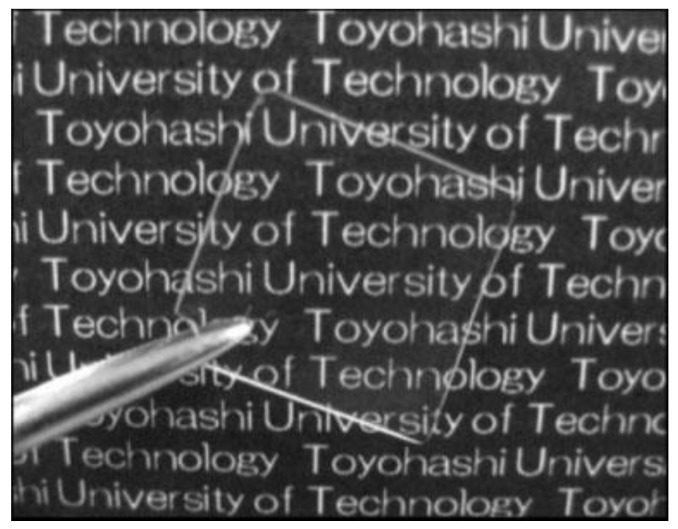
Photograph of a PVPA film derived from PDMVP prepared with *^t^*BuLi/*^n^*Bu_3_Al at −78 °C. Reprinted with permission from [77]. Copyright (2010) Wiley-VCH GmbH.

A similar approach to PVPA via anionic polymerization of CH_2_=CHP(O)(OMe)_2_ with subsequent hydrolysis was implemented by Takeichi et al. [77] with the use of *^t^*BuLi or *^t^*BuLi/*^n^*Bu_3_Al (1:5 mol/mol) as an initiator at the first stage (polymerization was conducted in toluene media), and conc. HCl with 12 h of reflux at the second stage. When polymerization was initiated by *^t^*BuLi/*^n^*Bu_3_Al instead of *^t^*BuLi alone, a threefold increase in monomer conversion was detected and, more significantly, *Ð*_M_ decreased by half, from 2.65 to 1.30, and isotacticity increased. The water solutions of the PVPAs were cast on a glass plate and allowed to stand at room temperature for seven days. After drying, the PVPA (*M*_n_ = 5.0 kDa, m = 52%), prepared through the radical polymerization, became viscous liquids, whereas PVPA (*M*_n_ = 5.5 kDa, m = 67%), prepared through the anionic process, formed self-standing transparent film. In this way, the quality of the PVPA obtained at –78 °C with the use of *^t^*BuLi/*^n^*Bu_3_Al initiator is *clearly*—in the proper sense of the word—illustrated by Figure 2.

Both free-radical and anionic polymerization of CH_2_=CHP(O)(OR)_2_ are usually not able to provide high monomer conversions and desirable molecular weight characteristics and microstructure of the polymer. In polyolefin chemistry, a similar problem was effectively solved by the use of coordination catalysis. However, conventional Ziegler-Natta and single-site catalysts, suitable for α-olefin polymerization, cannot be applied in the coordination polymerization of phosphonates due to the high electron donation ability of the oxygen atom of →P=O fragment. To date, only rare earth metal complexes were used successfully to polymerize dialkyl vinyl phosphonates.

**Scheme 15:**
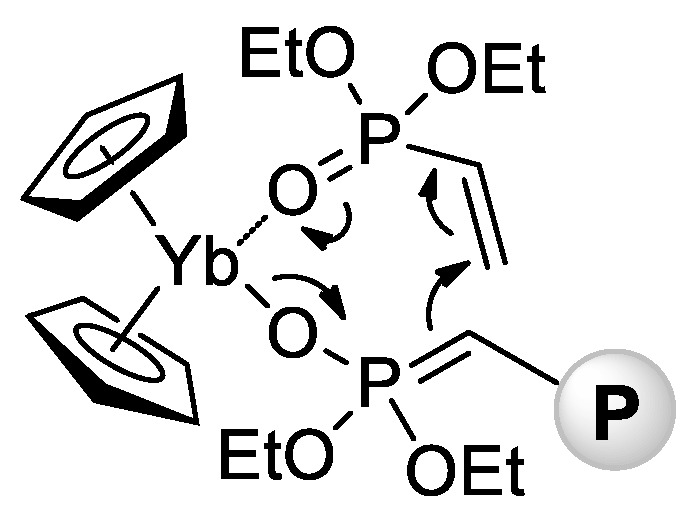
Postulated intermediate in the group-transfer mechanism of CH_2_=CHP(O)(OEt)_2_ polymerization, catalyzed by Cp_2_YbX. [79].

In 2010 [78], Rabe and Coll. reported that rare earth metal amides of the formula [N(SiHMe_2_)_2_]_3_(THF)_2_ (Ln = La, Nd, Sm) catalyze polymerization of CH_2_=CHP(O)(OEt)_2_ with a formation of high-MW isotactic polymers (*M*_n_ = 65–84 kDa, *Ð*_M_ = 3.1–3.6, mm = 66–79%). A corresponding Y complex was found to be inactive in polymerization, forming a stable adduct [N(SiHMe_2_)_2_]_3_[CH_2_=CHP(O)(OEt)_2_]_2_. In the same year, Rieger and Coll. described the first example of metallocene-catalyzed polymerization of CH_2_=CHP(O)(OEt)_2_ [79]. The complexes (η^5^-C_5_H_5_)_2_YbX (Cp_2_YbX, X = Cl, Me) have demonstrated high activities in toluene reaction media, polymerization had the ‘living’ character, and *M*_w_ up to 1280 kDa were achieved. Already in this first work, Rieger et al. proposed a group-transfer mechanism of polymerization (Scheme 15) that was confirmed and discussed in subsequent research articles [80,81,82,83,84,85,86] and reviews [87,88] of this scientific group.

**Scheme 16:**
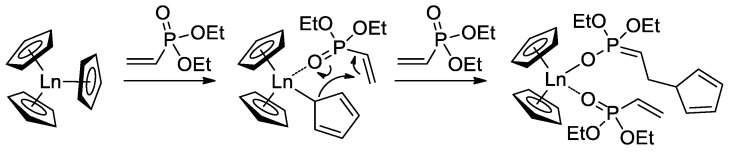
Initiation of the Cp_3_Ln-induced polymerization of CH_2_=CHP(O)(OEt)_2_ [80].

When using Cp_3_Ln complexes (Ln = Lu, Yb, Tm, Er, Ho, Dy) as coordination catalysts of CH_2_=CHP(O)(OEt)_2_ polymerization, C_5_H_5_ fragment acted as an initiator of the chain growth (Scheme 16) [80]. Polymerization had the ‘living’ character, polymers with given *M*_n_ (60–710 kDa depending on monomer-to-catalyst ratio) and *Ð*_M_ = 1.05–1.36 were obtained. Corresponding PVPAs were then prepared for the reaction with Me_3_SiBr, followed by mild hydrolysis (HCl/MeOH). Dimethyl and diisopropyl vinyl phosphonates were also polymerized. DCS studies have shown that CH_2_=CHP(O)(OR)_2_-based polymers decompose at 280–340 °C (R = Et) and 245–270 °C (R = ^i^Pr) with olefin elimination and formation of PVPA. When using Cp_3_Yb catalyst, highly statistic copolymers of CH_2_=CHP(O)(OR)_2_ (R = Me, Et, *^n^*Pr) were obtained [81].

**Figure 3:**
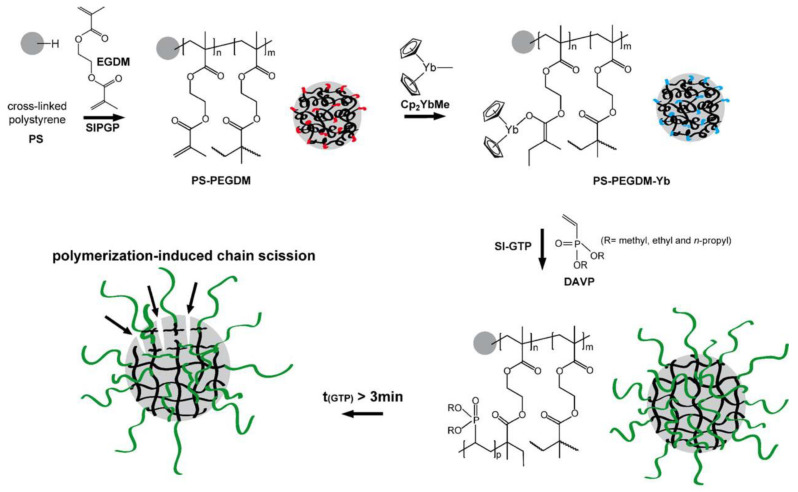
Schematic illustration of the covalent grafting of a PEGDM on a cross-linked polystyrene microsphere and subsequent immobilization of the Cp_2_YbMe. In the next step, SI-GTP of dialkyl vinyl phosphonate leads to the formation of poly(dialkyl vinyl phosphonate)-functionalized microspheres. Reprinted with permission from [90]. Copyright (2014) Wiley-VCH GmbH.

Since Cp_2_YbX-catalyzed polymerization of acrylates and CH_2_=CHP(O)(OEt)_2_ proceed according to similar group-transfer mechanisms, a surface-initiated group transfer polymerization (SI-GTP), which allows the covalent modification of solids with dense poly(vinylphosphonate) brushes, was carried out [89]. Silicon surfaces were modified with methacrylate functionalities, either via a self-assembled monolayer of 3–(trimethoxysilyl)propyl methacrylate or via a poly(ethylene glycol dimethacrylate) film (prepared by self-initiated photografting and photopolymerization), the surfaces were treated with Cp_2_YbMe, and then by vinyl phosphonate monomer. After Me_3_SiBr and Me_3_SiBr treatment, hydrophilic PVPA surfaces formed. A similar approach was realized using cross-linked polystyrene microspheres [90] (Figure 3).

**Figure 4:**
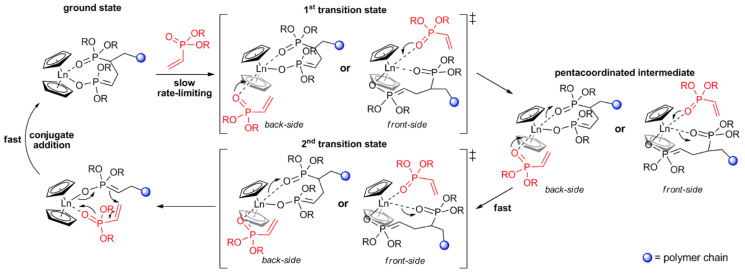
Elemental steps of rare earth-mediated group transfer polymerization of vinyl phosphonates. The rate-limiting step is an S_N_2-type associative displacement of the polymer phosphonate ester by a vinylphosphonate monomer, presumably via a pentacoordinated intermediate. Reprinted with permission from [82]. Copyright (2013) American Chemical Society.

As a result of the mechanistic studies of polymerization of CH_2_=CHP(O)(OR)_2_ [82] it was demonstrated that initiation by Cp_2_LnX follows a complex reaction pathway depending on the nature of X. For X = Me, CH_2_SiMe_3_ initiation occurs via the abstraction of the acidic α-CH of the vinylphosphonate, and for X = Cp, SR initiation occurs via the nucleophilic transfer of X to a coordinated monomer. For X = Cl, OR a monomer-induced ligand-exchange reaction with a formation of active Cp_3_Ln catalyst was observed. The further elemental steps of the reaction are presented in Figure 4 [82].

**Scheme 17:**
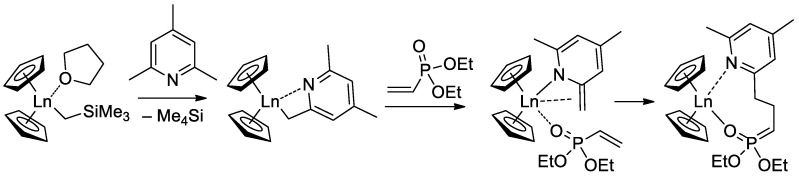
Synthesis of Cp_2_Ln(CH_2_(C_5_H_2_Me_2_N)) via C−H bond activation by σ-bond metathesis and initiation step of the CH_2_=CHP(O)(OEt)_2_ polymerization [83].

Since the initiation stage of Cp_2_LnX-mediated polymerization of CH_2_=CHP(O)(OR)_2_, this stage is significantly slower than propagation stages (leading to the broadening of MWD and partial loss of the polymer chain control), subsequent studies were focused on the development of highly efficient initiators. In that capacity, Cp_2_Ln derivatives of 2,4,6-trimethylpyridine were proposed [83]. The first stage of the process is presented in Scheme 17. Polymerization experiments have demonstrated the absence of the induction period when using this new initiator.

**Scheme 18:**
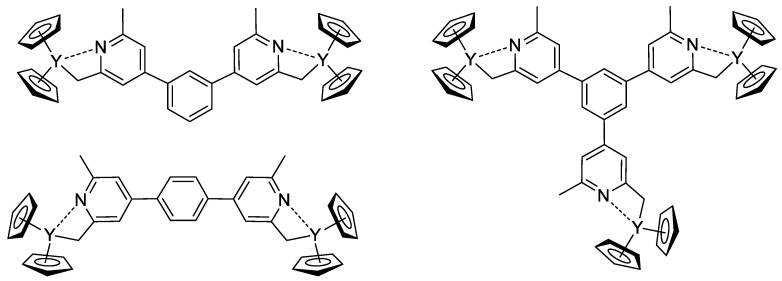
Efficient initiators for two- and three-pronged polymerization of CH_2_=CHP(O)(OEt)_2_ [85].

The concept of the efficient initiator of rare earth metal-mediated group transfer polymerization was beautifully realized by the example of the core-first polymer synthesis [85]. Based on 1,3- (or 1,4-bis-) and 1,3,5-tris(2,6-dimethylpyridin-4-yl)benzene, Cp_2_Y complexes (Scheme 18) were obtained and used as initiators of the group transfer polymerization of CH_2_=CHP(O)(OEt)_2_. When using dinuclear complexes, bimodal polymer distribution differing in molecular weight by a factor of two was detected. In the case of the trinuclear complex, trimodal molecular weight distribution was observed.

**Figure 5:**
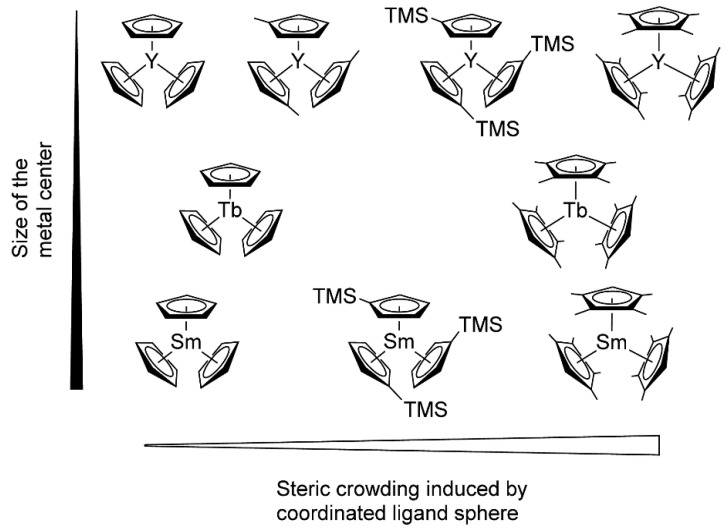
Molecular structures of monosubstituted and tetramethyl-substituted Cp_3_Ln catalysts studied in polymerization of CH_2_=CHP(O)(OEt)_2_. Reprinted with permission from [84]. Copyright (2016) American Chemical Society.

Rieger and Coll. also drew attention to another important aspect of lanthanidocene-initiated polymerization of CH_2_=CHP(O)(OR)_2_, namely, steric factors (Figure 5) [84]. As a result of the metal−ligand interactions, the studied catalysts varied in their properties, ranging from inert (Cp_3_Ln, Ln = Tb, Sm) to highly active ((C_5_H_4_Me)_3_Y. (C_5_Me_4_H)_3_Sm was the first example of the active ‘early’ lanthanidocenes, and in general, the activities accelerated with decreasing cation size. Furthermore, the polymerization behaviors of (C_5_Me_4_H)_3_Ln complexes differed from unsubstituted or monosubstituted metallocenes. The pentacoordinate intermediates of these complexes exhibited lengthened metal-monomer bond lengths, resulting in a higher enthalpy ΔH^≠^ term, and are partly compensated by lower ΔS^≠^ contributions to the activation barrier.

**Scheme 19:**
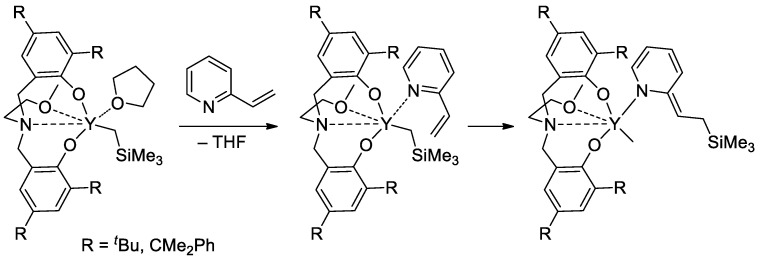
Six-membered initiation mechanism for 2-vinylpyridine and Y chelate complexes in polymerization of CH_2_=CHP(O)(OR)_2_ (R = Et, ^i^Pr) [91].

Rare-earth metal-based catalysts of the polymerization of vinyl phosphonates are not limited by sandwich lanthanide complexes. The high catalytic activity of the yttrium bis(phenolate) in the polymerization of CH_2_=CHP(O)(OR)_2_ (R = Et, ^i^Pr) was demonstrated [91], 2-vinylpyridine efficiently activated the pre-catalyst at the initiation stage (Scheme 19).

**Scheme 20:**
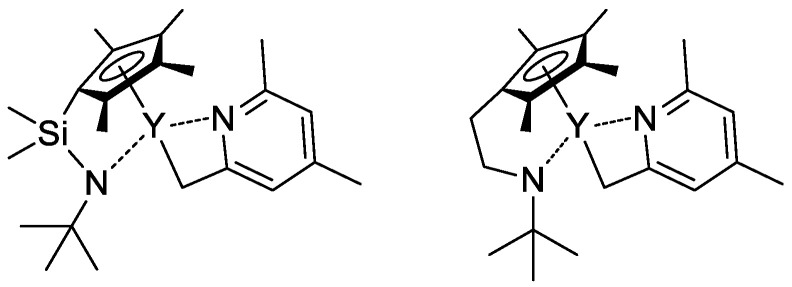
Efficient constrained-geometry catalysts for ‘living’ polymerization of vinyl phosphonates [86].

**Figure 6:**
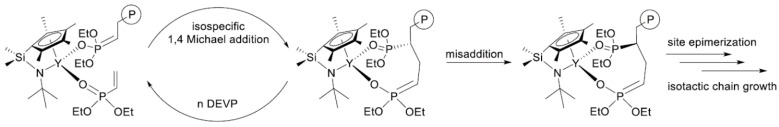
Proposed stereocontrol mechanism and formation of stereoerrors for the isospecific polymerization of CH_2_=CHP(O)(OEt)_2_. Reprinted with permission from [86]. Copyright (2019) American Chemical Society.

In their recent publication, Rieger and Coll. described the synthesis and catalytic behavior of half-sandwich ‘constrained geometry’ complexes of Y and Al in the polymerization of CH_2_=CHP(O)(OEt)_2_ [86]. The complexes of Y (Scheme 20) have demonstrated high activity even at −78 °C, under these conditions highly isotactic polymer (*mmm* > 98%) was obtained. The proposed stereocontrol mechanism is presented in Figure 6.

Note that in 2013 [92], Ni and Coll. have demonstrated high catalytic activity of Ln(BH_4_)_3_(THF)_3_ (Ln = Y, La, Nd, Sm, Gd, Dy, Lu) in group transfer polymerization of CH_2_=CHP(O)(OEt)_2_. ‘Living’ character of the reaction was confirmed by the termination of the polymerization with the use of PhCH_2_Cl or EtI, however, obtained polymers were characterized by broadened MWD (*Ð*_M_ = 1.63–1.81).

In conclusion, it is important to notice that the treatment with Me_3_SiBr, followed by mild hydrolysis, or multi-hour reflux in HCl is not the only method for transforming poly(phosphonates) to PVPA. High efficiency of the use of CF_3_SO_3_H or highly acidic cationites in ‘wet’ (hydrolysis) and ‘dry’ (olefin elimination) transformation of dialkyl phosphonates to phosphonic acids was demonstrated by Han and Coll. [93]. Different synthetic approaches to the hydrolysis of phosphinates and phosphonates have been reviewed very recently [94], and nothing prevents researchers from using these methods in the synthesis of PVPA and other sidechain PCPAs.

#### 2.3.2. Copolymerization of VPA and VPA Derivatives with Other Vinyl Monomers

Since VPA homopolymers have the prospective but limited potential of applications, valuable works on the synthesis of copolymers containing –CH_2_CHP(O)(OH)_2_– fragments were published even at the end of the last century.

**Scheme 21:**
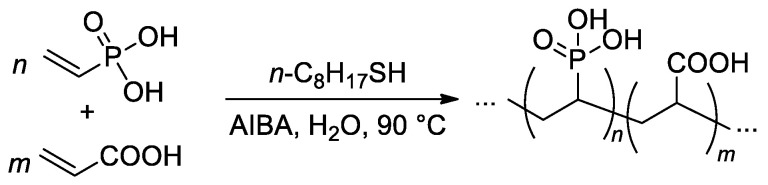
Free-radical copolymerization of acrylic acid and VPA [95].

The free-radical copolymerization of acrylic acid and VPA was conducted by Budd and Coll. in aqueous media (Scheme 21), AIBA was used as an initiator [95]. As the VPA content in the feed was increased, the monomer conversion and yield of the copolymers showed a general decrease. The reactivity ratios of acrylic acid (r_1_) and VPA (r_2_) were 4.09 and 0.042, respectively. Such a high difference in r_1_ and r_2_ values may give rise to composition drift, where the composition of the copolymer changes as the polymerization proceeds. Where the VPA content in the feed was below 50%, a chain transfer agent was introduced into the polymerization to restrict the molecular weight. Using this method, a range of copolymer compositions were produced with consistent molecular weights (*M*_w_ = 150–200 kDa) up to a VPA content of 59 mol%. At higher VPA contents, high-MW polymers were not obtained, and the highest *M*_w_ for VPA homopolymer was 29 kDa.

**Scheme 22:**
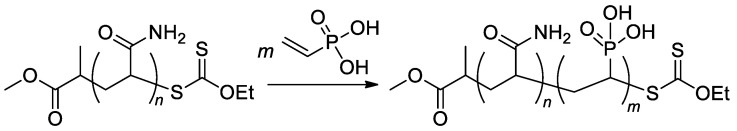
Aqueous RAFT/MADIX polymerization of VPA via chain extension of a polyacrylamide macroxanthate [96].

RAFT/MADIX approach was used in the synthesis of poly(acrylamide)-*b*-PVPA copolymers (Scheme 22) [96] with a given length of poly(acrylamide) block and varied length of PVPA fragment, copolymers with *M*_n_ 5.5–11.4 kDa were obtained.

When using ethylene glycol diacrylate, cross-linked copolymers of acrylic acid and VPA were obtained [97]. Copolymers of VPA and 1-vinyl-1,2,4-triazole with 1:1, 1:2, and 2:1 comonomer ratios were obtained in DMF solution at 85 °C with AIBN initiator, the *M*_n_ values ranged from 6.0 to 8.2 kDa, the dispersity *Ð*_M_ was ~2 [98]. Copolymerization of VPA and methacrylic ester of mPEG 475 was carried out in ethyl acetate with the use of AIBN as an initiator, side reactions and gel formation were detected [99].

UV-initiated copolymerization of VPA and acrylamide at the oxidized silicon surface in the presence of 2,2-dimethoxy-2-phenylacetophenone (DMPA) was described in [100], the binding of the copolymer with the surface was confirmed by photoelectron spectroscopy.

The synthesis of CH_2_=CHP(O)(OMe)_2_, CH_2_=CF_2,_ and CH_2_=CHSi(OEt)_3_ terpolymers was described in [101]. The best results were obtained when using 2,5-dimethyl-2,5-di(*tert*-butylperoxy)hexane as an initiator of the reaction that was conducted in dimethyl carbonate at 115 °C. Si–O–Si cross-linking was accompanied by partial hydrolysis of methyl phosphonate fragments.

Poly(styrene)-*b*-PVPA was obtained by *^n^*BuLi-initiated sequential anionic polymerization of styrene and CH_2_=CHP(O)(O^i^Pr)_2_ in THF media with subsequent hydrolysis [75].

The syntheses of VPA copolymers with 2-deoxy-2-methacrylamido-D-glucose, 4-acryloylmorpholine, or acrylamide, were the subject of the recent study of Nazarova and Coll. [102]. The reactions were conducted in DMF or MeOH (AIBN initiator) or aqueous media (AIBA initiator). VPA has demonstrated values of reactivity comparable with 4-acryloylmorpholine and acrylamide, thus confirming the preference for the use of VPA instead of VPA esters in copolymerization.

With the use of (NH_4_)_2_S_2_O_8_ initiated free-radical polymerization in aqueous media, random terpolymers of VPA, acrylonitrile, and methyl acrylate were synthesized [103]. Poly(vinylphosphonic acid-co-styrene-co-maleic anhydride) was obtained using AIBN as an initiator and DMSO as a solvent [104]. Water-soluble initiator was used in the random copolymerization of VPA with 5-(methacryloylamido)tetrazole [105].

As a final note, the use of rare-earth metal complexes in the synthesis of copolymers of vinyl phosphonates was the subject of the recent review of Rieger et al. [88].

#### 2.3.3. Homopolymerization and Copolymerization of Phosphorylated Acrylates

Derivatives of acrylic and methacrylic acid, containing –OP(O)(OH)_2_ and –P(O)(OH)_2_ fragments, have been evidently regarded as prospective monomers for the synthesis of sidechain PCPAs. There is extensive literature on this subject, and in this section, we have tried to present the most interesting and recent works in this field.

Evidently, one of the common methods of the polymerization of acrylates—anionic polymerization—is not applicable for derivatives of phosphoric and phosphinic acids, and the vast majority of research has used free-radical polymerization in one way or another. Trivial methods of free-radical polymerization (solution process, azo compounds, peroxides or UV exposure as initiators, no CTAs or other additives) are fully applicable to phosphorylated and phosphonated acrylates. This is particularly true in the case of acrylates, containing the phosphorus atom in an alkoxy fragment of the acrylate molecule (Scheme 4), due to the high reactivity of these monomers, and a number of examples of the homo- and co-polymerization of phosphorylated and phosphonated (meth)acrylates can be cited [22,23,25,26,28,30,31,37,38,39,106,107,108]. Note that the studies of AIBN-initiated copolymerization of methyl methacrylate and CH_2_=C(Me)C(O)OCH_2_P(O)(OMe)OH showed *r*_1_ and *r*_2_ values of 0.98 and 1.03, respectively, thus confirming close reactivity of ‘conventional’ and phosphonated acrylates [30].

**Scheme 23:**
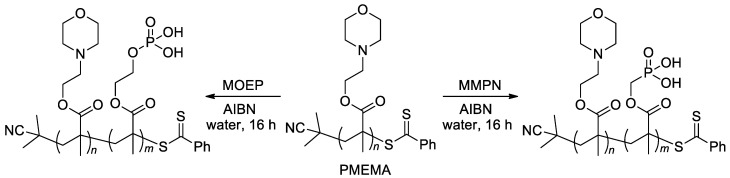
Synthesis of diblock copolymers via RAFT polymerization [29].

On the other hand, RAFT homo- and co-polymerization were successfully applied to similar acrylate monomers [29,33,109]. In particular, zwitterionic diblock copolymers were synthesized by copolymerization of the macro-RAFT agent, PMEMA (Scheme 23) with methacrylate monomers MOEP and MMPN via RAFT polymerization [29].

**Scheme 24:**
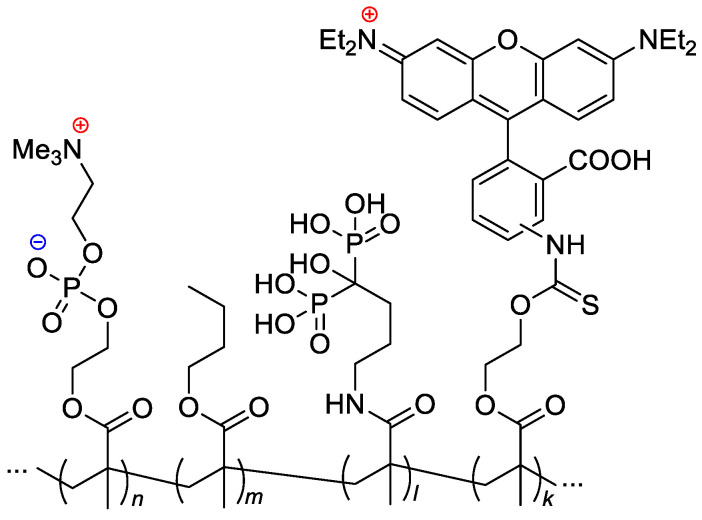
Formulation of the bone-targeting phospholipid copolymers [40].

Copolymers of MPC and methyl acrylate with (4-formylphenyl) fragment via –(OCH_2_CH_2_)_9_– spacer, were synthesized by RAFT polymerization [110]. AIBN-initiated copolymerization of MPC, *n*-butyl methacrylate, MADP (optionally) and rhodamine-linked methacrylate (optionally, for the pharmacokinetic studies) resulted in copolymers with *M*_n_ = 14–43 kDa and Ð_M_ ~2 [40] (Scheme 24).

The hyper-crosslinked polymer was obtained by AIBN-initiated free-radical polymerization of (CH_2_=C(Me)C(O)OCH_2_CH_2_O)_2_P(O)OH (BMEP) in DMF [111].

**Scheme 25:**
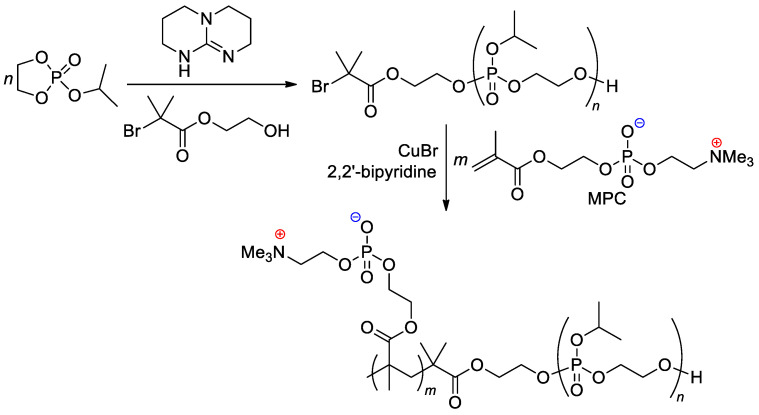
Synthetic route to block copolymer containing both main-chain and sidechain phosphate fragments [112].

The zwitterionic block copolymer was synthesized by sequential carrying out of organocatalytic ROP of 2-isopropoxy-1,3,2-dioxaphospholane 2-oxide (^i^PrOEP) with the use of BrCMe_2_C(O)O(CH_2_)_2_OH as an initiator, and atom transfer radical polymerization (ATRP) of the phosphorylcholine-substituted methacrylate (MPC) in the presence of CuBr and 2,2′-bipyridine (Scheme 25) [112]. Note that MOEP-based polymer brushes were obtained with the use of the ATRP approach, based on an SH-substituted –CMe_2_Br initiator bonded with gold nanoparticles [113].

**Scheme 26:**
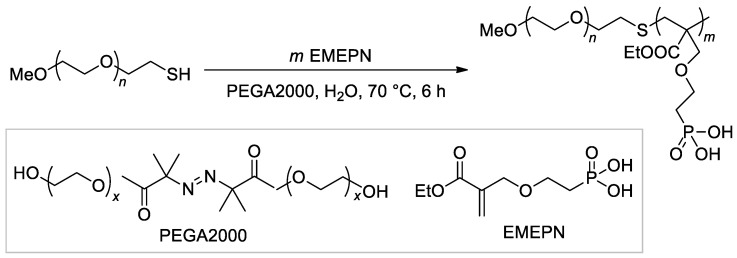
The use of ATRP with PEGA2000 initiator in the synnthesis of hydrophilic diblock-copolymer [114].

The synthesis of the diblock copolymer, containing mPEG-5000 fragment and polymer of (2-((2-(ethoxycarbonyl)allyl)oxy)ethyl)phosphonic acid as the second block [114], is of particular interest due to the chosen method, ATRP with thiol CTA and macroinitiator PEGA2000 (Scheme 26).

#### 2.3.4. Homopolymerization and Copolymerization of Other Vinyl Monomers

**Scheme 27:**
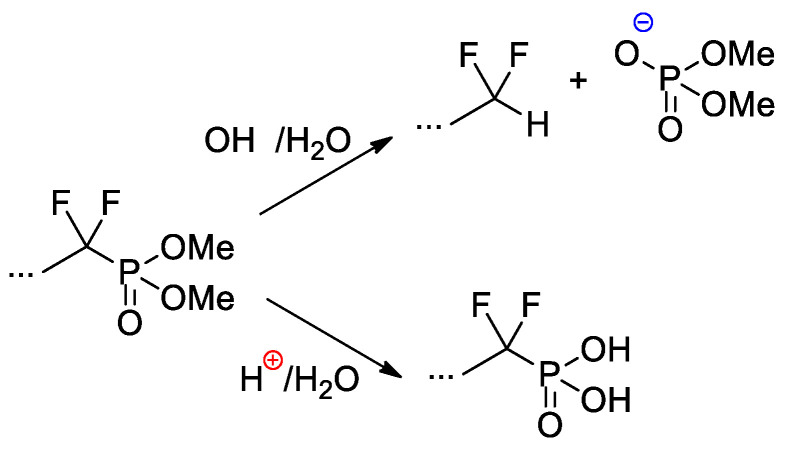
Hydrolysis of perfluoroalkyl phosphonate fragment [48].

Copolymers of CF_2_=CF_2_, CF_2_=CF(C_3_F_7_) and CF_2_=CFO(CF_2_)_3_P(O)(OMe)_2_ were obtained with the use of AIBN-initiated reaction in 1,1,2-trichloro-1,2,2-trifuoroethane (R-113) media [48]. These copolymers as such have not demonstrated outstanding characteristics, but, nevertheless, a very significant chemical aspect should be mentioned here. Whereas hydrolysis of –P(O)(OR)_2_-functionalized polymers is not accompanied by noteworthy side reactions, hydrolytic cleavage of the –CF_2_P(O)(OMe)_2_ fragment is not so straightforward (Scheme 27). Under basic conditions, the cleavage of the C–P bond was observed, but acidic hydrolysis resulted in the formation of the phosphinic acid.

For the synthesis of homopolymers of phosphonated styrenes SP and SbP (Scheme 9) and their block copolymers with poly(isobutylene) Park and Coll. used ATRP in toluene media with CuCl/N,N,N′,N″,N″-pentamethyldiethylenetriamine catalytic system [51]. Corresponding PCPAs were obtained by treatment with Me_3_SiBr/CHCl_3_ (36 h at 40 °C) followed by 8 h of methanolysis. Alter and Hoge [50] have synthesized –CH_2_CF_2_P(O)(OH)_2_-functionalized polystyrene (*M*_n_ = 12.1 kDa, *Ð*_M_ = 2.42) by AIBN-initiated polymerization of styrene containing—CH_2_CF_2_P(O)(OEt)_2_ substituent in the position 4, followed by two-stage deprotection (reaction with Me_3_SiBr/CH_2_Cl_2_ and methanolysis).

#### 2.3.5. Cyclopolymerization

**Scheme 28:**
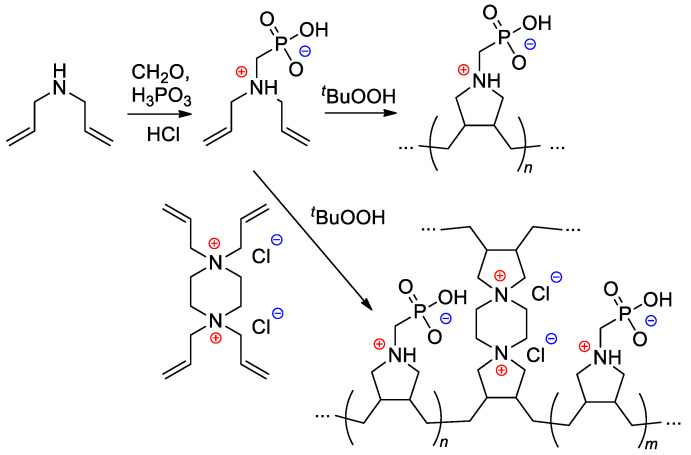
Synthesis of phosphonate-containing diene monomer and its free-radical diene cyclopolymerization [115].

Starting from diallylamine, both linear and cross-linked PCPAs were synthesized by Al Hamouz and Ali [115] by free-radical diene cyclopolymerization approach (Scheme 28). Treatment of the polymers by NaOH resulted in anionic polyelectrolytes that have an excellent adsorption capacity for metal ions (see Section 3.3).

### 2.4. Phosphonation and Phosphorylation of the Polymers

#### 2.4.1. Direct Phosphonation of ‘Saturated’ Polymers

The reaction of saturated hydrocarbons with PCl_3_ and molecular oxygen results in the phosphonation of some of the carbon atoms in paraffin [116]. This reaction is also applicable to polyolefins [117]. To a first approximation, saturated hydrocarbons form alkylphosphonyl chlorides by the overall reaction, presented in Equation (1),

R–H + 2PCl_3_ + O_2_ → R–P(O)Cl_2_ + POCl_3_ + HCl (1)

and the R–P(O)Cl_2_ can further be hydrolyzed to the corresponding phosphonic acid. This reaction requires an excess of PCl_3_ and is difficult to control. Bulk phosphonation negatively affects the mechanical characteristics of the polymers [117]. Twenty years ago, Allan and Coll. reported the results of the study of surface phosphonation of low-density polyethylene. The most uniform surface treatment was achieved in the gas phase at 25 °C within 30 min [118]. Further studies in this field have not been conducted, apparently, due to the low environmental friendliness of the process and the complexity of controlling the properties of the materials obtained.

#### 2.4.2. Modification of the Reactive –OH Groups in the Polymer Backbone

**Scheme 29:**
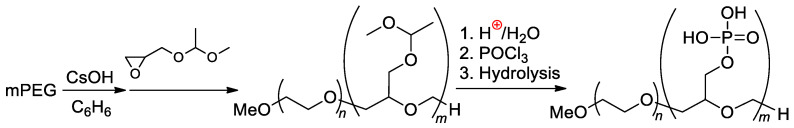
Synthesis and hydroboration of PEG-*b*-poly(buta-1,3-diene) to PEG-*b*-poly((2-hydroxyethyl)ethylene), followed by phosphorylation [122]. Original reaction scheme presented in [122], was corrected.

Double-hydrophilic copolymers containing nonionic PEG fragments and ionic phosphorylated polyglycidol blocks were synthesized by Penczek and Coll. [119,120] by the phosphorylation of the polymer obtained previously by mPEG-initiated polymerization of CH_2_=CHOMe-protected glycidol [121,122] (Scheme 29).

**Scheme 30:**
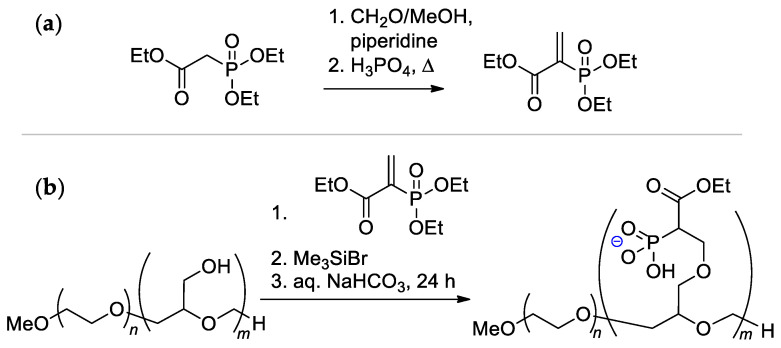
(**a**) Synthesis of ethyl 2-(diethoxyphosphoryl)acrylate; (**b**) Functionalization of the polyglycidol block [123].

The alternative method of functionalization of polyglycidol block is based on the reaction with ethyl 2-(diethoxyphosphoryl)acrylate, followed by hydrolysis (Scheme 30) [123]. The reaction of the –OH groups with ethyl 2-(diethoxyphosphoryl)acrylate did not require catalysts and was completed at 25 °C in THF or CH_2_Cl_2_ after 1 day. Such an approach has an advantage due to the hydrolytic stability of the C–P bond, but the issue of the toxicity of copolymer remains open.

**Scheme 31:**
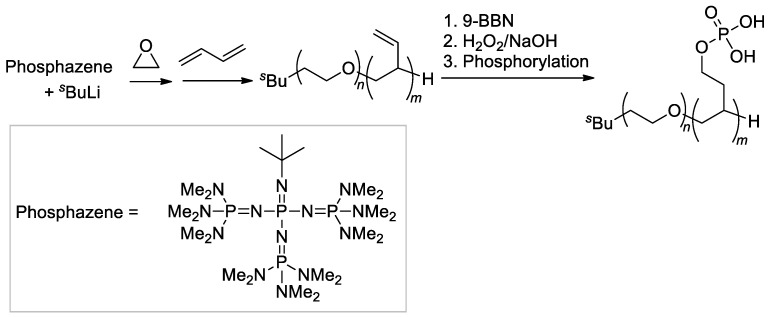
Synthesis and hydroboration of PEG-*b*-poly(buta-1,3-diene) to PEG-*b*-poly((2-hydroxyethyl)ethylene), followed by phosphorylation [122]. Original reaction scheme, presented in [122], was corrected.

Another example of double-hydrophilic block copolymers, containing PEG fragment and hydrocarbon chain with –CH_2_CH_2_OP(O)(OH)_2_ substituents, was also synthesized by Penczek’s group [122] by post-modification of the block copolymer of oxirane and buta-1,3-diene (Scheme 31), obtained previously using ^s^BuLi/phosphazene initiator [124]. Note that this strategy was reported later by Dimova and Coll. [125] who described the same method for the synthesis of block copolymers.

However, one should not forget about an even simpler and more accessible polymer, poly(vinyl alcohol). Its phosphorylation was carried out by the reaction of poly(vinyl alcohol) (*M*_w_ = 76 kDa) with ~60% aq. H_3_PO_4_ (1 h, reflux), the product was separated using precipitation in MeOH [126]. Degree of phosphorylation was not determined in this work.

#### 2.4.3. Modification of the Reactive –COOH Groups in the Polymer Backbone

Since (co)polymers of acrylic and methacrylic acid are synthetically available, their post-modification via chemical binding of –OP(O)(OH)_2_ and –P(O)(OH)_2_ groups with carboxylate fragment seems feasible and promising. Efficient transformation of –COOH to –C(OH)[P(O)(OH)_2_]_2_ fragment under the action of PCl_3_/H_3_PO_3_, followed by hydrolysis, was proposed by Kieczykowski et al. in 1995 [127]. This approach was used in the synthesis of partially phosphorylated PEG-*b*-poly(methacrylic acid) (PEG-*b*-PMMA-PO_3_H_2_(1%)) [128].

#### 2.4.4. Modification Based on Michaelis–Arbuzov Reaction

**Scheme 32:**
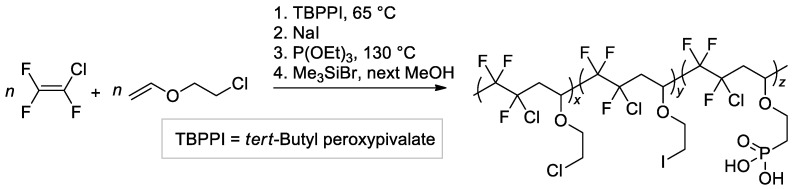
Synthetic pathway to graft phosphonic acid groups onto fluorinated copolymers obtained by radical copolymerization of CF_2_=CFCl with 2-chloroethyl vinyl ether [129].

The copolymer of CF_2_=CFCl with 2-chloroethyl vinyl ether of the altering microstructure was obtained by free-radical copolymerization and modified with the use of the Michaelis–Arbuzov reaction [129] (Scheme 32). Five copolymers with different phosphonic acid content were thus synthesized.

Functionalization of poly(styrene-ethylene/butylene-styrene) block copolymer (*M*_n_ = 89 kDa, *Ð*_M_ < 1.06) was carried out in two stages, by chloromethylation and the subsequent Michaelis–Arbuzov reaction with P(OEt)_3_ followed by acidic hydrolysis [130]. Copolymer samples with 9, 24, 42, and 54% degrees of chloromethylation/phosphonation were obtained for subsequent preparation of the proton conducting membranes (see Section 3.4).

**Scheme 33:**
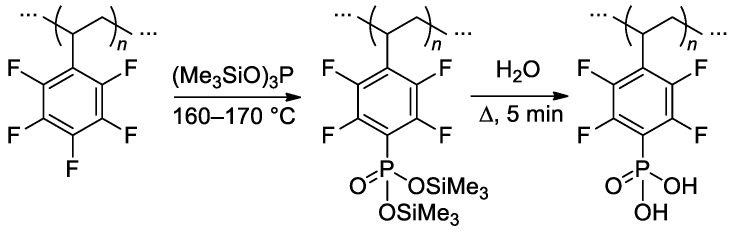
Direct selective phophonation of poly(pentafluorostyrene) [131].

Efficient (up to 90%) post-phosphonation of poly(pentafluorostyrene) (*M*_n_ = 30.2 kDa, *Ð*_M_ = 1.92) was achieved via the Michaelis–Arbuzov reaction with (Me_3_SiO)_3_P (2.4–3 equiv.) in bulk at 160–170 °C, followed by hydrolysis [131] (Scheme 33). In [132], the phosphonation protocol was modified for achieving a lower degree of phosphonation (lower (Me_3_SiO)_3_P to poly(pentafluorostyrene) ratio, 100 °C, homogenization by the use of N,N-dimethylacetamide). The materials obtained allowed us to make significant progress in the development of high-temperature fuel cells [133,134,135] (see Section 3.4).

**Scheme 34:**
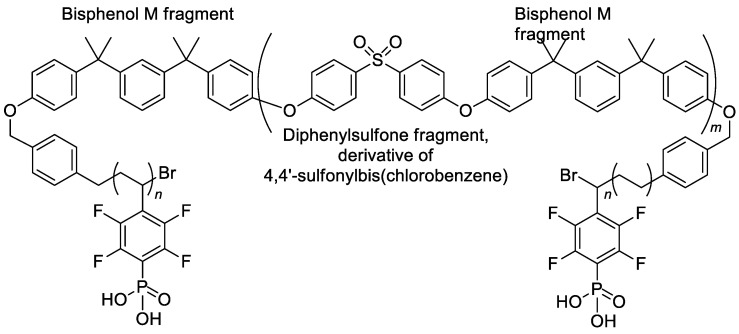
Structure of phosphonated triblock copolymers [136].

The reaction, presented in Scheme 33, was also used for post-modification of the complex triblock copolymer, the product of the (i) polycondensation of Bisphenol M with 4,4′-sulfonylbis(chlorobenzene); (ii) treatment with 1,4-bis(bromomethyl)benzene; (iii) ATRP with pentafluorostyrene [136]. The structural formula of the copolymer thus obtained is presented in Scheme 34.

#### 2.4.5. Other Methods

**Scheme 35:**
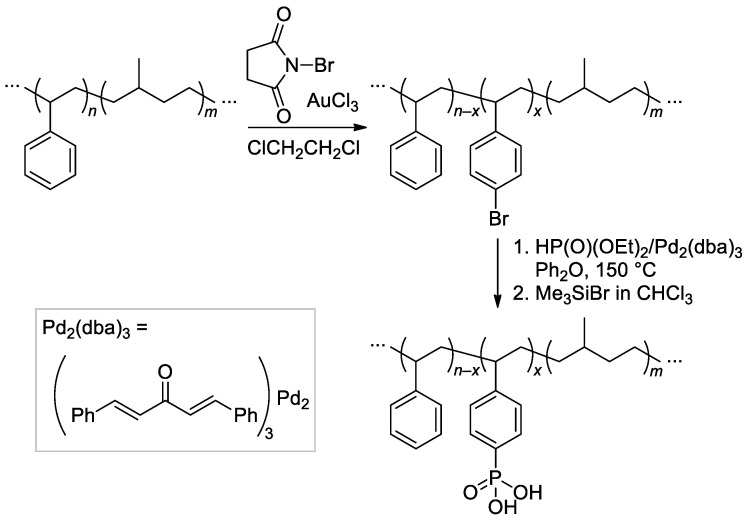
Synthetic procedure for phosphonated poly(styrene-*b*-methylbutylene)s [137,138].

Poly(styrene-*b*-methylbutylene)s were synthesized by sequential anionic polymerization of styrene and isoprene, followed by selective hydrogenation of aliphatic C=C bonds. Bromination, cross-coupling reaction with HP(O)(OEt)_2_, and two-stage cleavage (Scheme 35) resulted in phosphonated copolymers with different content of the –P(O)(OH)_2_ groups [137,138].

**Scheme 36:**
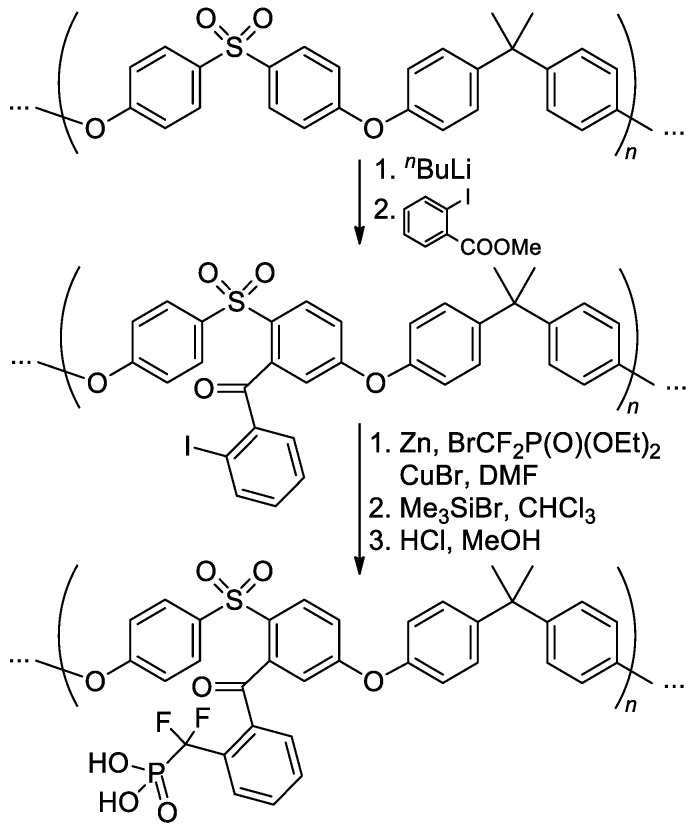
Synthetic pathway to o-benzoyl(difluoromethylenephosphonic acid)-functionalized polymer [139].

Cross-coupling approach was also used in the post-modification of the Bisphenol A/4,4′-sulfonylbis(chlorobenzene) polycondensation product [139] (Scheme 36).

The original approach to cross-linked copolymers, derivatives of poly(vinylidene difluoride), proposed by Sinirlioglu et al. [140], is based on dehydrofluorination of –(CH_2_CF_2_)_n_– with a formation of unsaturated fragments, free radical-initiated graft copolymerization with glycidyl methacrylate, and the reaction of the oxirane fragments with PVPA at the final stage. Polymer membranes were obtained by evaporation of the polymer solutions in DMF.

**Figure 7:**
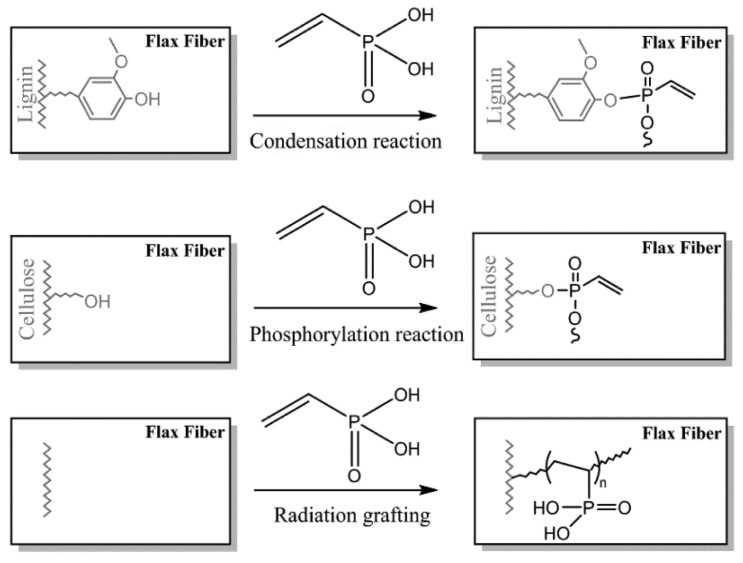
VPA grafting reactions on flax fibers through chemical modification in soft conditions (top), chemical modification in severe conditions (middle), and radiation grafting (bottom). Reprinted with permission from [141]. Copyright (2018) Elsevier B. V.

Two routes for grafting of the VPA on flax fabrics (81 wt% of cellulose, 13 wt% of hemicelluloses, and 2.7 wt% of lignin) were studied in [141], namely, radiation grafting and chemical modification (Figure 7). These approaches were studied in order to improve flame retardant characteristics of the fibers, qualitatively different results were obtained for the methods used (see Section 3.5.1).

Hydrolyzed polyvinyl alcohols (72 and 145 kDa) were phosphorylated using reactions with H_3_PO_4_/urea, with POCl_3_ and subsequent hydrolysis, and with (MeO)_3_P/I_2_ [142]. In the latter case, PCPAs were obtained by the reactions with NaI in N-methyl-2-pyrrolidone or via conventional silylation/methanolysis.

Efficient mechanochemical phosphorylation of cellulose, lignin, PEG, poly(vinyl alcohol), and poly(vinyl chloride) using P_2_O_5_ was described in [143]. Phosphate loadings of ~0.2 kg∙mol^−1^ were achieved for PEG and lignin, whereas for cellulose, poly(vinyl alcohol), and poly(vinyl chloride) phosphate loadings were 3.3, 4.4, and 2.2 kg∙mol^−1^, respectively.

### 2.5. Other Synthetic Approaches to SideChain PCPAs

#### 2.5.1. Metathesis Polycondensation

**Scheme 37:**
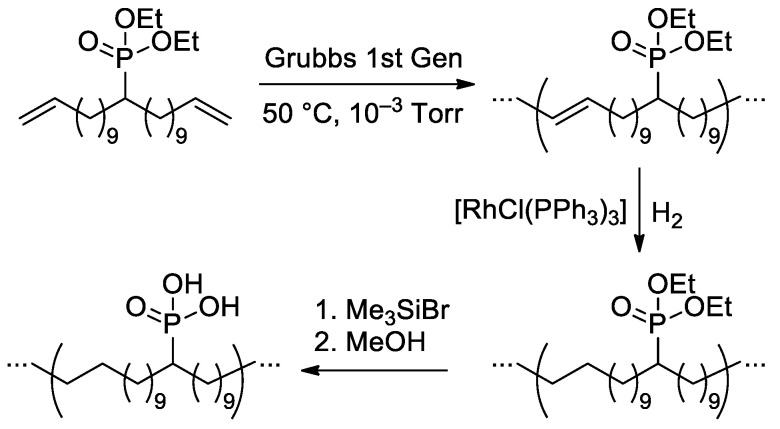
Synthetic scheme of precisely functionalized polyethylene with phosphonic acid on every 21st carbon [144].

Polyethylene’ containing one –P(O)(OH)_2_ substituent in 21 carbon atoms, was synthesized in three steps by Wagener and Coll. [144] (Scheme 37). The authors noted that compared to the typical oxidative phosphorylation reactions on polyethylene [118], the proposed method offers complete control of the microstructure, which will lead to a systematic understanding of how microstructure and resulting morphology dictate particular properties. However, the properties of the material obtained were not studied in depth due to the low solubility of the –P(O)(OH)_2_ substituted PE (before deprotection, the *M*_n_ was 19.5 kDa, and *Ð*_M_ was 1.7).

Later, the Wagener group expanded a range of diene monomers by varying the methylene units between phosphonate and –CH=CH_2_ fragments and a number of –P(O)(OR)_2_ groups at the central carbon atom [145]. As a result, a series of diethyl phosphonate substituted ‘polyethylenes’ with different lengths of (CH_2_)_n_ spacers between –CHP(O)(OEt)_2_– or –C[P(O)(OEt)_2_]_2_– fragments were obtained, without further transformation to PCPAs.

#### 2.5.2. Ring-Opening Metathesis Polymerization

**Scheme 38:**
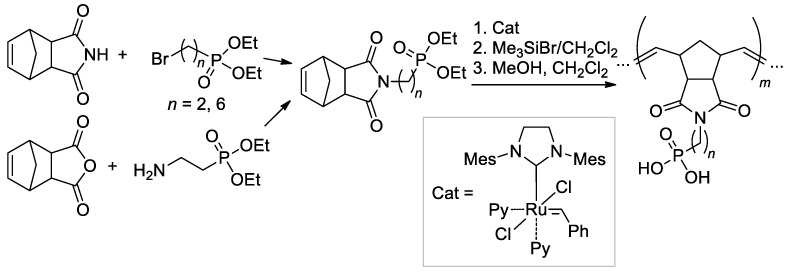
Synthesis of norbornene-based phosphonate monomers and ROMP with a formation of –P(O)(OH)_2_-functionalized polymers [146].

An efficient method of the synthesis of the phosphonated polymers with a well-defined MWD, composition, and architecture was proposed by Bingöl and Coll. [146] based on synthetically available adducts of cyclopenta-1,3-diene with maleimide or maleic anhydride (Scheme 38). Block- and stat-copolymers were also obtained with the use of *n*-Bu substituted imide. Note that –P(O)(OH)_2_-functionalized polymers were obtained by treatment with Me_3_SiBr in dry CH_2_Cl_2_ to yield TMS esters, followed by cleavage in a MeOH/CH_2_Cl_2_ mixture.

#### 2.5.3. Nucleophilic Polycondensation

**Scheme 39:**
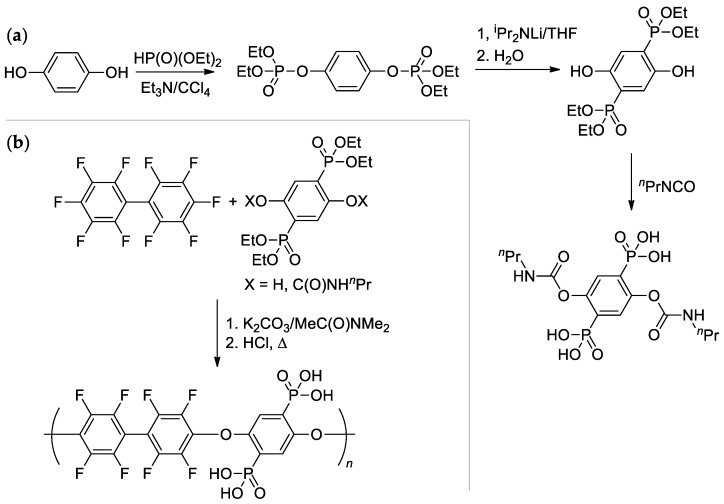
(**a**) Synthesis of diphosphonated hydroquinone and its dicarbamate derivative; (**b**) Polycondensation with perfluoro-1,1′-biphenyl with a formation of high-MW copolymer [147].

The high reactivity of the C–F bond in the *p*-position of RC_6_F_5_ was also exhibited in the reaction of perfluoro-1,1′-biphenyl with diphosphonated hydroquinone in the presence of K_2_CO_3_ [147] (Scheme 39). Copolymer with *M*_n_ = 28.4 kDa was obtained; intermediate modification of (2,5-dihydroxy-1,4-phenylene)diphosphonic acid by the reaction with *^n^*PrNCO resulted in dicarbamate derivative, more efficient in polycondensation (milder conditions, *M*_n_ = 37.9 kDa).

#### 2.5.4. Reductive Coupling

**Scheme 40:**
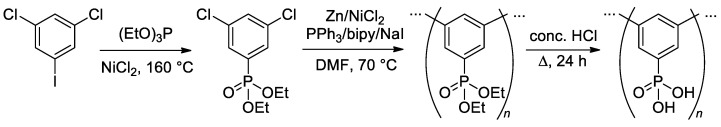
Synthesis of poly(1,3-phenylene-5-phosphonic acid) (PmPPA) [148].

Nickel-catalyzed reductive coupling in the presence of metallic zinc was used in the synthesis of a prospective polymer, poly(1,3-phenylene-5-phosphonic acid) (PmPPA) [148]. During the synthesis of PmPPA (Scheme 40), –P(O)(OEt)_2_ group was introduced by nickel-catalyzed Arbuzov reaction of 1,3-dichloro-5-iodobenzene with triethyl phosphite, and after reductive coupling phosphonate groups were hydrolized by HCl.

## 3. Properties and Applications of Sidechain PCPAs

### 3.1. Physico-Chemical Characteristics and Solution Behavior of SideChain PCPAs

#### 3.1.1. Physical State and Mechanical Properties

The physical state and characteristics of sidechain PCPAs can vary widely due to the diversity of the structures of macromolecules. Obviously, no one canceled the common patterns of the influence of the polymer microstructure on physicomechanical characteristics, but non-selective methods of the synthesis of sidechain PCPAs (mostly free-radical polymerization, see Section 2) make irrelevant the issue of polymer tacticity. Discussions about the influence of the polymer microstructure on polymer properties make sense only for well-researched polymers, synthesized by different methods, and PVPA is actually the only polymer in the group under consideration.

Depending on the molecular mass value, the samples of regioregular and atactic PVPA, obtained by free-radical polymerization, represent viscous liquid [77] or amorphous solid [66,149]. The sample morphology drastically changed with increasing water content. For example, for a relatively high-MW polymer (*M*_w_ = 62 kDa) up to ~45% relative humidity (RH) (~17% water sorption), the material remained a white powder. Above 45% RH, a transparent film was formed. With further increasing humidity, this film became sticky and turned into a gel at about 100% RH [149]. In the amorphous PVPA, no melting transition was detected, and a glass transition at ~220 °C was masked by dehydration with a formation of phosphonic acid anhydride [75] (on other data, for low-MW amorphous PVPA the glass temperature *T*_g_ = 141 °C [150]).

The behavior of cross-linked copolymer of HEMA, MOEP (0–100 mol% relative to HEMA), and BMEP (0.5 wt%) (see Scheme 4, obtained by γ-initiated free-radical polymerization, [108]), when immersed in water was found to be dependent on the fraction of MOEP in the polymer. The polymers with 0–20 mol% MOEP did not fracture during swelling and displayed concentration-dependent water sorption, while the copolymers with greater than 30 mol% MOEP exhibited catastrophic fracturing during swelling, resulting in the destruction of the sample geometry. In this way, with high content of acidic groups, even cross-linking does not ensure the stability of the copolymer shape (and this should be considered in developing formulations of hydrogels and composites).

#### 3.1.2. Solution and Colloidal Behavior

The solution behavior of sidechain PCPAs mainly depends on the nature of the polymer backbone and the relative number of phosphonate (or phosphate) fragments. For example, PVPA is soluble in water and ethanol and can be purified by precipitation of the saturated ethanol solution in ethyl acetate [69]. Highly phosphonated PmPPA is soluble in water and water/alcohol mixtures but poorly soluble in pure methanol [148].

A high similarity of the solution behavior of poly(acrylic acid) and PVPA was demonstrated by Wegner and Coll. [151]. The results of the more recent study of the solution behavior of PVPA and random VPA/CH_2_=CHP(O)(OMe)_2_ copolymers by SEC-MALLS measurements [73] are presented in Table 1. The coefficients calculated from MHS plots were between 0.5 and 0.8 meaning that randomly coiled polymers were formed.

**Figure 8:**
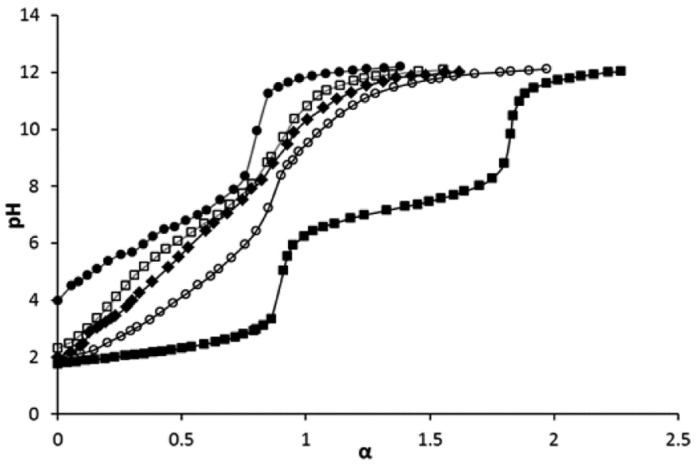
pH titration curves of VPA (■), PVPA-0 (●), PVPA-30 (□), PVPA-60 (♦), and PVPA-100 (○), (co)polymer concentration was 1 mg∙mL^−1^ for all cases, α is nominal degree of neutralization. Reprinted with permission from [95]. Copyright (2016) American Chemical Society.

As demonstrated by Wegner et al. [57], while the VPA monomer shows the two dissociation steps expected, PVPA behaves as a monoprotic acid. The results of the study of copolymers of VPA and acrylic acid by potentiometric titration are presented in Figure 8 [95]. The nominal degrees of neutralization α of the poly(acrylic acid) (PVPA-0), copolymers containing 30 and 60 mol% of VPA (PVPA-30 and PVPA-60, respectively), poly(VPA) (PVPA-100) were calculated by taking into account elemental analysis data. The results of this study were in line with [57,65] with respect to PVPA. As opposed to the homopolymer, the titration curves of the copolymers showed two neutralization steps, which may be attributed to the contributions of VPA units with a pK_a1_ = 2.49 (step 1) and acrylic acid unit with a pK_a2_ = 7.74 (step 2).

In contrast with PVPA, for PmPPA (see Scheme 40) the pH titration curves showed distinct pH steps for the addition of one and two equivalents of base [148].

The solution behavior of polymers affects the ability to use certain methods of the preparation of polymer films and scaffolds. Among other approaches, ES molding has been successfully used in obtaining fibrous materials for different applications [152,153]. However, this method remains practically unused for sidechain PCPAs, despite their polyelectrolyte nature. Only the works of Lee [154,155] and Akar et al. [103] reported the results of the studies on the subject. In [154], ES fibrous mats with good morphology were prepared from an aqueous solution of the poly(vinyl alcohol) (PVA)/PVPA mixture. To prevent swelling, the samples were subjected to thermal treatment or chemical cross-linking (methanol/glutaraldehyde). In the next work [155], ES fibrous materials with lower content of PVPA were prepared and stabilized by heating at 150 °C for 24 h *in vacuo*, chemical cross-linking was not used to avoid toxicity. In [103], the preparation of the ES fibrous materials from a copolymer of VPA, acrylonitrile, and methyl acrylate is described, and copolymer solutions in DMF were used.

Amphiphilic diblock copolymers usually demonstrated complex solution behavior. For example, PMEMA-*b*-poly(MOEP) and PMEMA-*b*-poly(MMPN) (for their synthesis, see Scheme 23) in basic aqueous media formed core-shell micelles as the PMEMA block formed insoluble micelle core. It is noteworthy that PMEMA-*b*-poly(MMPN) diblock copolymers interacted strongly with Ca^2+^ with a formation of reverse micellar structures. [29].

As was shown in [110], complex random copolymer, containing phosphorylcholine and doxorubicin fragments can self-assemble into nanoparticles with doxorubicin as the core, and hydrophilic P(MPC-co-PEGMA-BZ, see Section 3.3.4) as the shell in an aqueous solution.

### 3.2. Metal Complexation of the Sidechain PCPAs, Polymer-Inorganic Hybrids, and Composites

#### 3.2.1. Complexation of Sidechain PCPAs with Metal Ions

The reaction of PVPA with calcium tetraphosphate (Equation (2)) in 2:3 molar ratio results in the formation of Ca-PVPA and HAp [150].

2PVPA + 3Ca_4_(PO_4_)_2_O → 2Ca-PVPA + Ca_10_(PO_4_)_6_(OH)_2_ (2)

Cross-linked polymers presented in Scheme 28 demonstrated excellent adsorption of Pb^2+^ and Cu^2+^ ions. The values of the maximum adsorption capacity *Q*_m_ were 7.19 and 17.0 mmol∙g^−1^, respectively [115]. Another cross-linked copolymer was obtained from a terpolymer of MOEP, HEMA and 2-hydroxypropane-1,3-diyl bis(2-methylacrylate) by radical polymerization with acrylamide and N,N′-(propane-1,3-diyl)diacrylamide to mimic aquatic caddisworm silk and natural processes of Ca^2+^ complexation [106]. The clear homogeneous hydrogel was formed, and in the presence of divalent metal ions, Mg^2+^, Ca^2+^, and Zn^2+^, the hydrogels shrank to about 65% of the initial volume and became translucent. Above a critical phosphate sidechain density, hydrogels equilibrated with Ca^2+^ or Zn^2+^ ions displayed increased initial stiffness, strain-rate dependent yield behavior, and required 100 times more work to fracture than hydrogels equilibrated with Mg^2+^ or Na^+^ ions. The toughness of the bio-inspired hydrogels exceeded the toughness of cartilage and meniscus, suggesting potential application as prosthetic biomaterials, according to the authors of the study [106]. Mg^2+^ complexation by MOEP/HEMA copolymer in aqueous media was the subject of a separate study by the same research team [23].

Results of the study of VPA homopolymer and VPA copolymers with acrylamide and vinylsulfonic acid have demonstrated a high potential if the use of VPA copolymers as antiscalants for (Fe, Mg) silicates [156], the best efficiency was detected for a copolymer of VPA with vinylsulfonic acid (1:5 comonomer ratio).

The product of the hydrolytic cross-linking of the CH_2_=CHP(O)(OMe)_2_, CH_2_=CF_2,_ and CH_2_=CHSi(OEt)_3_ terpolymer exhibited very high Eu^3+^ extraction from its aqueous solutions [101]. Hyper-cross-linked homopolymer of (CH_2_=C(Me)C(O)OCH_2_CH_2_O)_2_P(O)OH demonstrated adsorption capacity for UO_2_^2+^ up to 800 mg∙g^−1^ and excellent selectivity for U(VI) adsorption over coexisting ions. In addition, this polymer also displayed high adsorption capacities for Gd, Sm, Ce, Nd, Tb, and Eu [111].

The ability of the –P(O)(OH)_2_ fragment to chemical binding with the metal ions largely determines the use of phosphates as anticorrosive agents. Copolymers of CF_2_=CH_2_ with CH_2_=C(CF_3_)C(O)OCH_2_P(O)(OH)_2_ (79–96 mol% of CF_2_=CH_2_, *M*_n_ up to 10 kDa) were synthesized by *tert*-amyl peroxy-2-ethylhexanoate initiated free-radical copolymerization in dimethyl carbonate [36]. Steel plates, coated with the copolymer, displayed satisfactory anticorrosion properties under a simulated seawater environment. An original and efficient approach to development of anticorrosive agents was proposed by Kousar and Moratti [157], via the use of methacrylate copolymers containing –(CH_2_)_2_(CF_2_)_7_CH3 and –CH_2_P(O)(OH)_2_ substituents. These copolymers formed monolayers at the stainless-steel surface (the contact angle >128°) and were stable after being submerged in water for a week. The best set of characteristics was achieved for copolymers containing phosphonated and fluorinated monomers in a 2:1 ratio.

#### 3.2.2. Effects of the Sidechain PCPAs on Crystal Growth and Morphology

**Figure 9:**
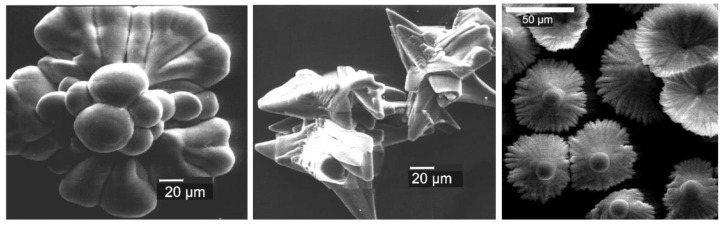
Examples of CaCO_3_ microcrystals obtained in the presence of PEG-*b*-(poly[2-(2-hydroxy ethyl)ethylene] with variable degrees of phosphate substitution. Reprinted with permission from [122]. Copyright (2002) Wiley-VCH GmbH.

A number of works from the Penczek group were devoted to the study of the synthesis and morphology of CaCO_3_ particles obtained in the presence of sidechain PCPAs, and an amazing diversity of the crystallite’s forms was observed [119,120,122,158,159] (Figure 9).

**Figure 10:**
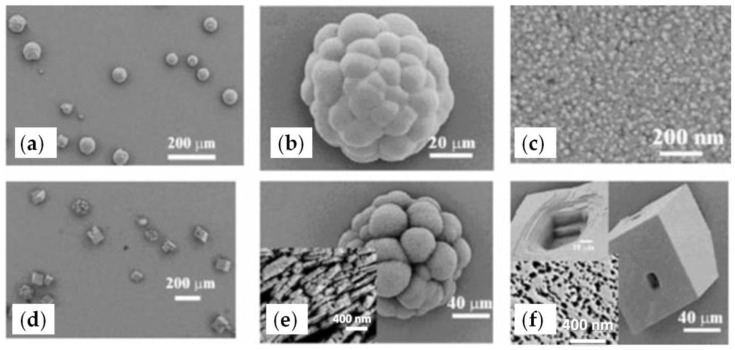
SEM images of CaCO_3_ obtained on a glass slip by the gas diffusion reaction after 1 week in 3 mL of solution with 10 mmol/L of Ca^2+^ in the presence of different concentrations of phosphonated copolymer. (**a**) 10^−2^ g∙L^−1^; (**b**) A typical spherical structure in image (**a**); (**c**) The surface structure of particle (**b**); (**d**) 10^−3^ g∙L^−1^; (**e**) one modified spherical structure in image (**d**) and the surface structure (inset); (**f**) one calcite rhombohedral structure in image (**d**) with a hole and the porous surface structure in the hole (inset). Reprinted with permission from [114]. Copyright (2005) Wiley-VCH GmbH.

Diblock copolymer mPEG-*b*-poly(EMEPN) (see Scheme 26) [114] demonstrated an interesting concentration effect on the formation of CaCO_3_ particles (Figure 10). Cölfen and Antonietti [160] have made a certain contribution to this issue; however, a detailed comparative analysis of their studies is complicated by the lack of detailed information on the structure of copolymers used.

Concentration effects, observed by the Penczek group, can be interpreted in view of the study of Dimova and Coll. [125] of the interaction of double hydrophilic block copolymers (see Scheme 31) with calcite crystals by isothermal titration calorimetry. It was shown that the interaction of copolymers with relatively large CaCO_3_ crystals is an exothermic process (well fitted by a Langmuir adsorption model). In the absence of large crystals, the observed endothermic process was attributed to polymer interaction with small clusters or aggregates of calcium carbonate of the type (CaCO_3_)_x_(H_2_O)_y_ where 1 < x < 100. The formation of similar aggregates cannot but affect the crystallization process in the presence of PCPAs. Morphological control of BaSO_4_ microstructure by copolymers PEG-*b*-PMMA and PEG-*b*-PMMA-PO_3_H_2_(1%) was studied by Cölfen and Coll. [128].

#### 3.2.3. Hybrid Nanoparticle Formation by Sidechain PCPAs

The ability of divalent transition metal ions to induce micellization by mixing aqueous solutions of the partly ionized poly(acrylamide)-*b*-PVPA copolymers (see Scheme 22) and metal ions was studied as a function of the nature of the metal cation [96]. Based on DLS measurements, the authors concluded that the micellization efficiency followed the sequence: Ni^2+^, Co^2+^ < Zn^2+^ < Mn^2+^ < Cu^2+^.

**Figure 11:**
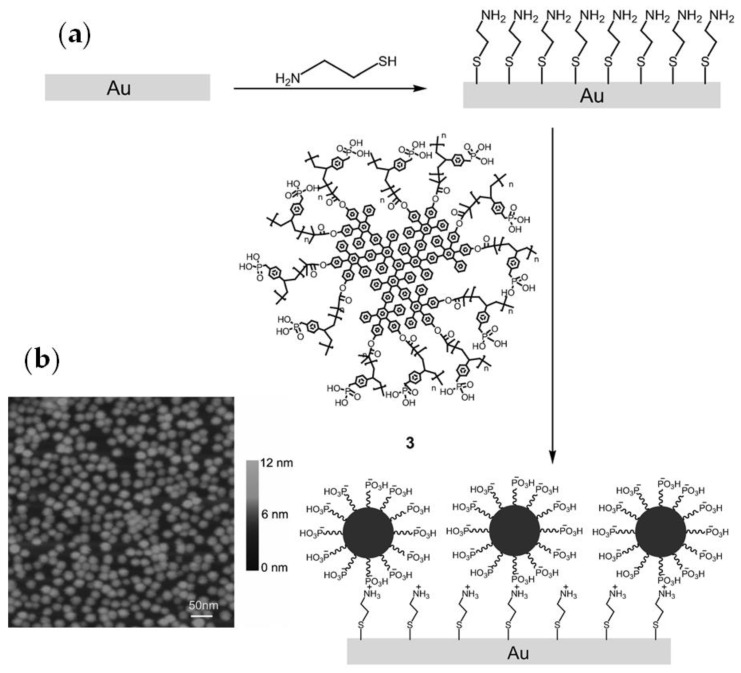
(**a**) The deposition of core-shell macromolecule **3** on a modified gold substrate through electrostatic interaction; (**b**) AFM image of a self-assembled film of **3** on gold. Reprinted with permission from [74]. Copyright (2012) Wiley-VCH GmbH.

After treatment of the gold surface by HS(CH_2_)_2_NH_2_, the self-assembled film (SAF) of the core-shell copolymer three (Figure 11a) was studied [74]. The self-assembly of a core-shell macromolecule three on gold was characterized by atomic force microscopy (AFM). As can be seen in Figure 11b, separated globular particles appear to be uniformly monodisperse with a diameter of ~20 nm.

When considering and using PVPA as a biomimetic analog of matrix phosphoproteins, Tay and Coll. [60] studied mineralization of cross-linked collagen in the presence of Ca^2+^, PO_4_^3−^, OH^−^ ions and poly(acrylic acid). Intrafibrillar and extrafibrillar mineralization via a bottom-up nanoparticle assembly based on the non-classical crystallization pathway were identified. Selected area electron diffraction patterns of highly mineralized collagen fibrils were nearly identical to those of natural bone, with apatite crystallites preferentially aligned along the collagen fibril axes. Conversely, only large mineral spheres with no preferred association with collagen fibrils were observed in the absence of PVPA.

#### 3.2.4. Polymer-Inorganic Composites

Composites of the Ca-PVPA and HAp were prepared by warm-pressing powder mixtures of Ca_4_(PO_4_)_2_O and PVPA at a weight ratio of 3.5:1 at temperatures of up to 300 °C and pressure up to 690 kpsi within 1 h [150]. The highest achieved values for the tensile strength and elastic modulus were 53 MPa and 32 GPa, respectively. The tensile strength values of the composites were lower than those of long bones (121–149 MPa), in the range of those reported for dentin (29.6–105.5 MPa), and higher than that reported for enamel (10.3 MPa). Elastic moduli of the composites were higher than long bones (17.2–18.6 GPa) and dentin (0.26–14.7 GPa) but lower than those of enamel (84.1–130 GPa). No further biomedical studies for these composites were conducted.

Attempt to prepare ferrimagnetic PVPA/BaFe_12_O_19_ nanocomposite was not entirely successful due to the adsorption of PVPA anions during the preparation of the nanocomposite that strongly influenced the magnetic properties, resulting in much lower saturation magnetization values [63].

A number of composite samples were prepared from phosphorylated poly(vinyl alcohol) and nano-sized HAp (the mean crystallite size 19 nm, 0–60 wt% loaded in composite formulation) [126]. In composites, obtained by chemical methods (with the use of an aqueous polymer solution), the phosphorylated polymer has demonstrated dispersant properties. With an increase in the content of HAp in composite, the tensile strength goes through the maximum of 26.2 MPa (50 wt% of HAp), Young’s modulus increases from 151 MPa (o% HAp) to 668 MPa (60% HAp), and elongation at break decreases from 39.2 to 4.8%.

### 3.3. Biomedical Applications of Sidechain PCPAs

#### 3.3.1. Sidechain PCPAs and Cell Viability/Metabolism and Differentiation

For sidechain PCPAs, hydrolytic degradation should lead to the formation of –OH functionalized polymers with unpredictable properties. Research in this area is fragmentary; however, the results of the effect of PCPAs and their metabolites on cell adhesion and proliferation showed that sidechain PCPAs rarely demonstrate toxicity.

In particular, when studying ES mats based on PVPA/PVA, no toxicity was detected in experiments with MG-63 osteoblast-like cells for untreated materials, but cross-linked membranes (MeOH/glutaraldehyde) turned out to be toxic, the cell proliferation was also suppressed significantly [154]. In the follow-up study [155], PVPA/PVA ES mats demonstrated low cytotoxicity with respect to M3TCT3-E1 preosteoblast cells, which increased with the increase in PVPA content in the composite. At the same time, the presence of PVPA facilitated cell proliferation.

**Figure 12:**
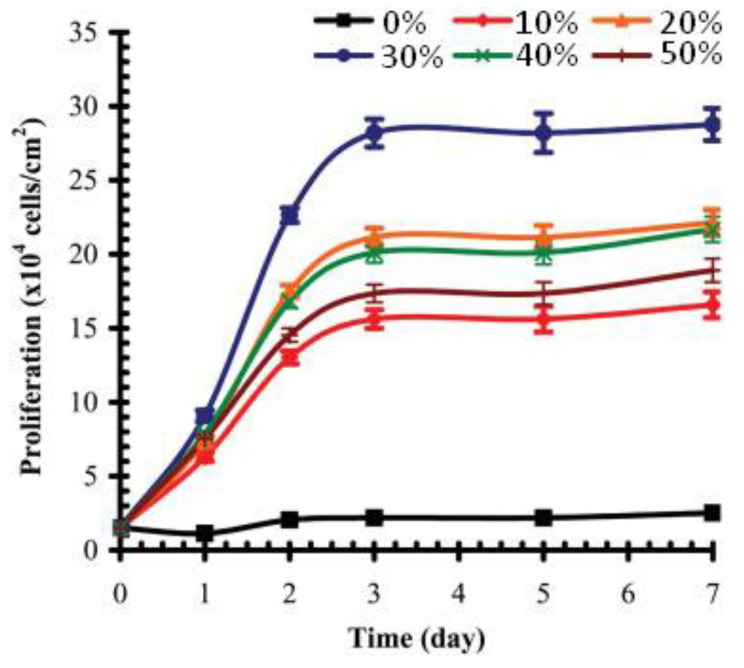
Number (proliferation) of MC3T3-E1 subclone 4 preosteoblast cells present on substrates modified with graft copolymers created from feed compositions of increasing VPA. Surfaces were modified with polyacrylamide (pVPA0), PVPA (pVPA100), and 10% (pVPA10), 20% (pVPA20), 30% (pVPA30), 40% (pVPA40), and 50% (pVPA50) VPA in the comonomer feed. Reprinted with permission from [100]. Copyright (2006) Wiley-VCH GmbH.

When studying osteogenic MC3T3-E1 subclone 4 cells on the surface of silicon, grafted by VPA—acrylamide copolymers, Gemeinhart and Coll. have demonstrated that cell adhesion and proliferation have a clear maximum that corresponds to 30 mol% content of VPA in the comonomer feed [100] (Figure 12). MC3T3-E1 maturation began at about the 20th day and was defined by matrix calcification and increased alkaline phosphatase activity. The mineralization of PVPA-modified surfaces was potentially due to both cellular differentiation and polymer-based calcification, but experiments in the absence of cells did not show mineralization. In this way, the cell-mediated mineralization, observed for the pVPA30-grafted surface, would benefit from osteointegration, which demonstrates high prospects for the use of VPA copolymers in the further design of bone tissue engineering scaffolds.

**Figure 13:**
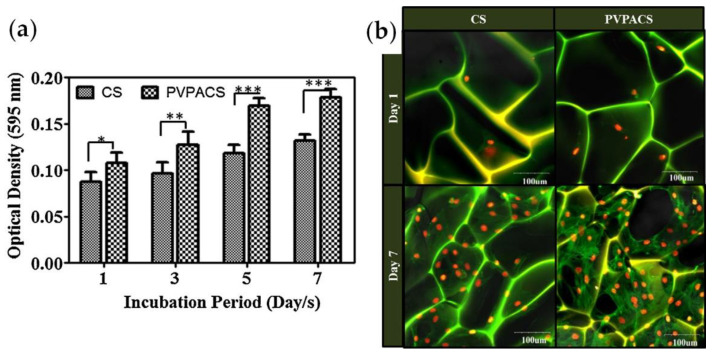
(**a**) MTT assay of MC3T3-E1 pre-osteoblast cells proliferation on CS and PVPACS matrices after 1, 3, 5 and 7 days of incubation period, data represent the mean ± SD forfour replicates and significantly different values (*** *p* < 0.001, ** *p* < 0.01, * *p* < 0.05); (**b**) Confocal images of MC3T3-E1 pre-osteoblast cells cultured on CS and PVPACS matrices after 1 and 7 days of the incubation period. Reprinted with permission from [161]. Copyright (2014) Elsevier B. V.

PVPA/chitosan composites were prepared by simultaneous adsorption of PVPA and N-(3-dimethylaminopropyl)-N′-ethylcarbodiimide mediated cross-linking; the porous three-dimensional matrices were prepared from the 2% aqueous solutions of chitosan (CS) or composite (PVPACS) containing 1% AcOH by thermally induced phase separation at −80 °C followed by sublimation of ice crystals [161]. MTT results for MC3T3-E1 pre-osteoblast cells have shown a higher degree of proliferation for PVPACS matrices compared with CS matrices (Figure 13).

Copolymers of acrylic acid, VPA, and ethylene glycol diacrylate as a cross-linking agent formed hydrogel. Freeze-drying the hydrogel resulted in obtaining the mesoporous materials. The pore diameter and formation of an interconnected pore network depended on the VPA content [97]. The experiments with human osteosarcoma-derived osteoblast (SaOS-2) cells showed that an increase in the VPA content supports cell adhesion and proliferation. The authors have said that hydrogels with 30 or 50 mol% VPA are ideal for use as bone void fillers, providing high swelling and increased osteoblast-like cell attachment and proliferation.

#### 3.3.2. Biocompatibility of Sidechain PCPAs and Prospects for Use in Bone Surgery

**Figure 14:**
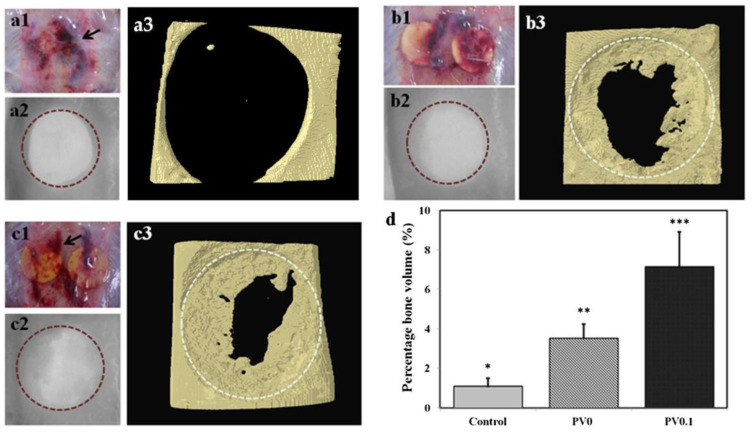
Comparison of defects without implant (control) (**a**), with PV0 (**b**) and PV0.1 (**c**). Images shown were obtained during extraction after 4 weeks of implantation (**1**), scanned micrograph from micro-computed tomography (**2**) and reconstructed three-dimensional structure of the defects (**3**). Blood clots (black arrows) may have resulted from the trauma of the surgery because the presence in the control (**a1**). Percentage bone volume was also computed, showing statistically higher values obtained in defects implanted with PV0.1 compared with nonimplanted defects (*** *p* < 0.001, ** *p* < 0.01, * *p* < 0.05) (**d**). Reprinted with permission from [155]. Copyright (2014) Wiley-VCH GmbH.

The effect of PVPA in addition to in vivo biocompatibility of PVPA/PVA ES mats was preliminarily investigated by the implantation of the PV0.1 membrane (1 wt% PVPA) in a defect compared with a defect with PV0 implanted (without PVPA) and with a defect without an implant (control) [155] (Figure 14). The results showed that the in vitro cell growth behavior on the PVPA-added membrane resulted in favorable tissue growth in vivo (confirmed by micro-CT results and histological studies). The histological observations derived from the rat skull defect implanted with PV0.1 also showed that the membrane was still intact after 4 weeks of grafting. The authors concluded that the efficiency of tissue growth is due to the phosphate groups, which facilitate the bonding process of the implant to the native host tissue.

**Figure 15:**
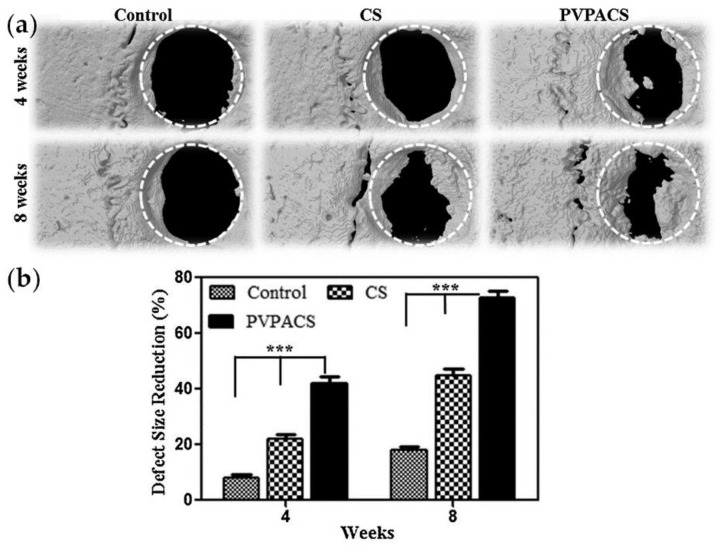
(**a**) Reconstructed three-dimensional images of μCT scans taken after 4 and 8 weeks of matrix implantation in rat calvarias; (**b**) Percentage size reduction quantified within standard ROI placed concentrically over the defect site from each μCT scan and time point. Asterisks define significant differences (*** *p* < 0.05). Reprinted with permission from [161]. Copyright (2014) Elsevier B. V.

PVPA/chitosan composite porous three-dimensional matrices were used for the implantation of full-thickness calvarial bone defects (diameter of 5 mm) in Sprague Dawley male rats [161]. Bone formation was limited by CS-treated groups, commencing only from the periphery of the host bone. However, with PVPACS treated group, bone formation was markedly higher, and some initiated in the center of the defect after 4 and 8 weeks of implantation (Figure 15). The osteoinductive effect of PVPA was also confirmed by hystological studies.

A complex porous composite material, based on poly(lactic-co-glycolic acid) microspheres, the copolymer of fumaric acid and oligo(ethylene glycol), and BMEP (see Scheme 4) as a cross-linker, was studied as a bone substituent after treatment with BMP-2 [162]. It was demonstrated that it is the presence of BMEP that enhance bone formation in the context of BMP-2.

#### 3.3.3. Sidechain PCPAs and Composites for Dental Applications

Dental self-etch adhesives (SEAs) are widely employed to adhere a restorative material to a tooth. Since the functionality of SEAs is provided by polymer formation, these *de facto* monomeric compounds are also discussed in this section.

**Figure 16:**
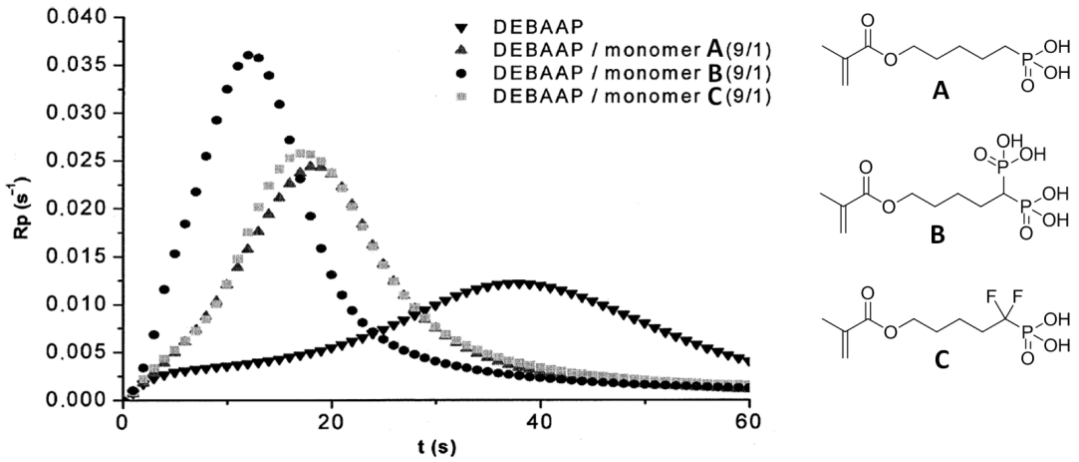
(Co)polymerization rate (Rp) as a function of time for DEBAAP and monomers A–C. Reprinted with permission from [34]. Copyright (2012) Elsevier B. V.

Le Pluart and Coll. [34] studied three different phosphonic, bisphosphonic, and difluoromethylphosphonic acid methacrylate monomers (see Scheme 7 and structures A–C in Figure 16).

The main objective of their study was to evaluate the influence of the nature of the acidic group on the reactivity and adhesive properties of the monomers. Photopolymerization of N,N′-diethyl-1,3-bis(acrylamido)propane (DEBAAP) with monomers A–C demonstrated higher reactivity of B, the difference between A and C was minimal. Dentin shear bond strength measurements have shown that primers based on B and C are significantly more efficient than the ones based on A. The dentin shear bond strength (SBS) values were 15.4, 19.7, and 20.6 MPa for A, B, and C, respectively. Therefore, monomers B and C appear to be great candidates for light-cured adhesive formulations.

**Figure 17:**
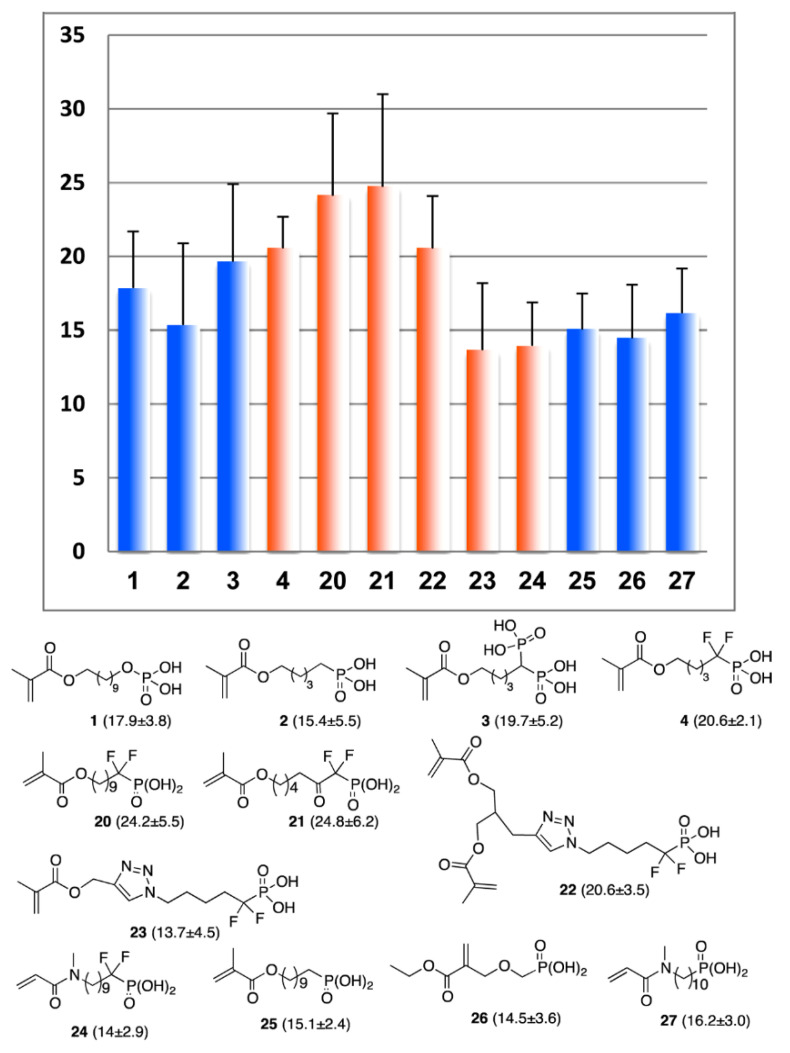
Mean shear bond strength (SBS) values (MPa) and standard deviation (MPa) for fluorophosphonate acids SEP, SBS values for fluorine-containing methacrylates are highlighted with orange. Reprinted with permission from [35]. Copyright (2014) American Chemical Society.

In further studies [35], new monomers with different spacers between methacrylate and –CF_2_P(O)(OH)_2_ fragments were synthesized (see Scheme 7) and used for the preparation of the primers with N,N-diethyl-1,3-bis(acrylamido)propane (10 mol%), containing photoinitiator (camphorquinone), coinitiator (ethyl 4-(dimethylamino)benzoate) and stabilizer (2,6-di-*tert*-butyl-4-methylphenol). Primers, coupled with the AdheSE bonding resin [163], were used to generate a bond between a standard Z100 A3 composite (3M/ESPE) and dentin. The results of the SBS measurements are presented in Figure 17. Dentin SBS measurements showed that –CF_2_P(O)(OH)_2_ containing primers are significantly more efficient than the –CH_2_P(O)(OH)_2_ containing methacrylates. Of particular note is the influence of the –(CH_2_)_n_– spacer length: the use of the SEP containing monomer **20** (n = 10, Figure 17) resulted in an SBS value of 24.2 MPa, whereas widespread monomers **1** and **25** had values of 17.9 and 15.1 MPa, respectively, and monomer **4** (n = 4) was characterized by SBS value of 20.6 MPa.

#### 3.3.4. Drug Delivery and Drug Release with the Use of Sidechain PCPAs

**Scheme 41:**
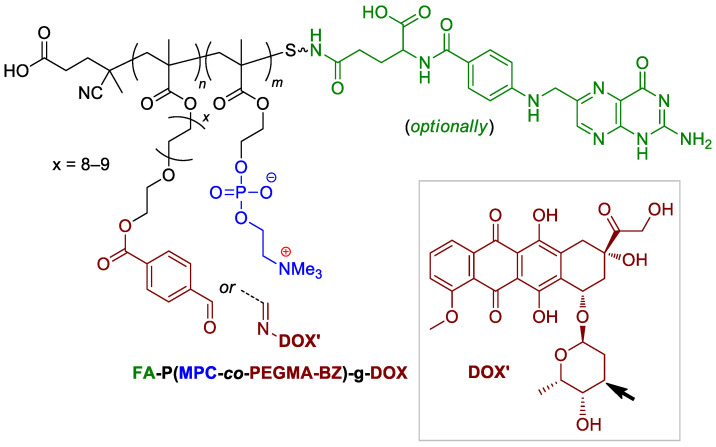
Structure of MPC-based copolymer for doxorubicin delivery [110].

Since zwitterionic polymers have excellent properties of hydration, anti-bacterial adhesion, long-term circulation and suppress nonspecific protein adsorption in vivo, Ni and Coll. described a novel folate-targeted and acid-labile polymeric prodrug under the microenvironment of tumor cells, based on the use MPC (Scheme 4) comonomer [110]. Functionalized copolymers containing the fragment of folic acid were able to chemical binding with doxorubicin (see Scheme 1 and Scheme 41), and the presence of phosphocholine fragments provided the delivery and release of the drug that was bonded with the neighboring fragment. The copolymers have demonstrated marked colloidal behavior with the critical aggregation concentration ~50 mg∙L^−1^. Nanoparticles were stable at pH 7.4 and tended to aggregate at a pH of 5.0. Colloid stability correlated with the drug release rates (after 98 h, 80 and 15% of DOX released at pH 5.0 and 7.4, respectively). The DOX-free copolymer was found to be non-toxic for normal cells (L929) and cancer cells (HeLa HepG2), whereas DOX-bonded copolymers have demonstrated a marked antitumor efficacy (IC_50_ of 0.76 mg∙L^−1^ and 0.62 mg∙L^−1^ after 48 h of incubation for PMPD and FA-PMPD, respectively), but were inferior to doxorubicin.

**Figure 18:**
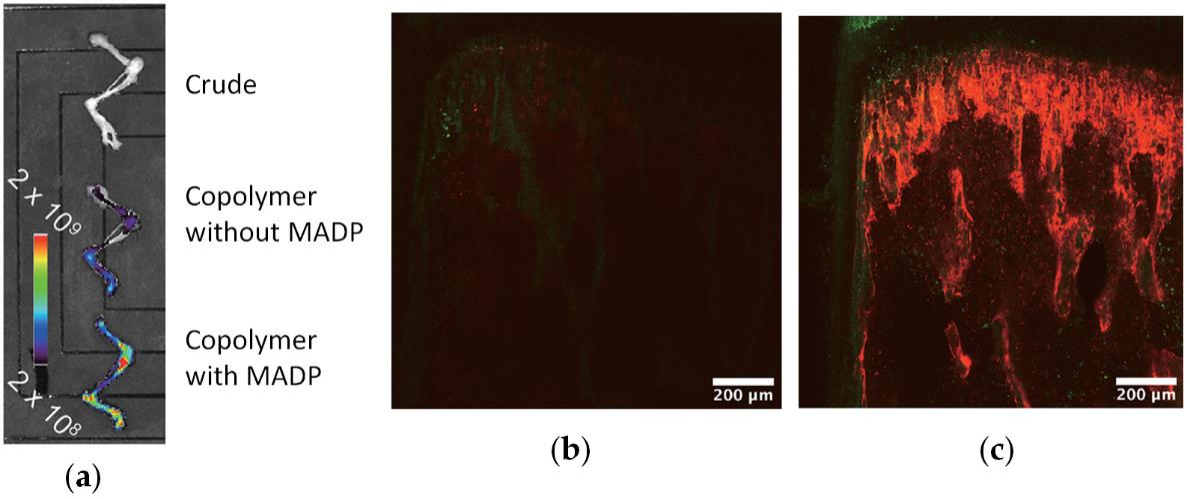
(**a**) An ex vivo fluorescence image of leg bone; (**b**,**c**) Confocal microscopy of the sagittal section of the proximal end of the tibia, harvested from mice 24 h after intravenous injection of copolymer/docetaxel for copolymer without and with MADP monomer units, rhodamine label was excited at 561 nm. Reprinted with permission from [40]. Copyright (2020) Wiley-VCH GmbH.

Given that current chemotherapies have limited effectiveness in eliminating bone metastasis, Iwasaki and Coll. [40] made a separate study to provide regional chemotherapy for this metastatic tumor. A bone-targeting drug carrier was designed by introducing the osteotropic bisphosphonate alendronate units into an amphiphilic phospholipid polymer (see Scheme 24). The copolymer formed nanoparticles (d < 30 nm), and diphosphonate units were exposed to the outer layer of the particles. These particles were found to be able to encapsulate a hydrophobic anticancer drug docetaxel. The complex formation did not hamper the pharmacological effect of the drug against several breast cancer cell lines. The fluorescence observations evaluated by an in vivo imaging system and fluorescence microscopy showed that the addition of diphosphonate units to the polymer–drug complex enhanced bone accumulation (Figure 18).

### 3.4. Polyelectrolytes Based on Sidechain PCPAs

Proton conduction in solid-state polyelectrolytes has aroused considerable interest in recent years in connection with the development of fuel cells, batteries, and water electrolyzers [164]. Sidechain PCPAs are regarded as prospective materials due to the presence of acid groups and the stable polymer backbone. Over the past decades, the number of research papers on the issue of sidechain PCPAs-based polyelectrolytes has significantly increased, and in this section we will look at some representative examples, taking into account the molecular structure of the polymers.

For polymer electrolytes, the critical structural variable is the concentration of the ionic functional groups that affect the water uptake and ion conductivity, expressed by the ion exchange capacity (IEC, molar equivalents of ion exchange group per g of dry polymer (mequiv∙g^−1^ or mmol∙g^−1^). Because –P(O)(OH)_2_-functionalized compounds are weaker acids than –SO_3_H-substituted polymers, phosphonic acids have lower conductivity in the presence of water. As a result, low-temperature electrochemical devices typically use sulfonated polymer electrolytes. However, for electrochemical devices operating at 100 °C and higher temperatures, phosphonic/phosphoric acid systems have an advantage because of their ability to provide proton conductivity in the absence of water. So in addition to proton conductivity, the high thermal stability of the polymers is desirable.

#### 3.4.1. PVPA-Based Polyelectrolytes

**Figure 19:**
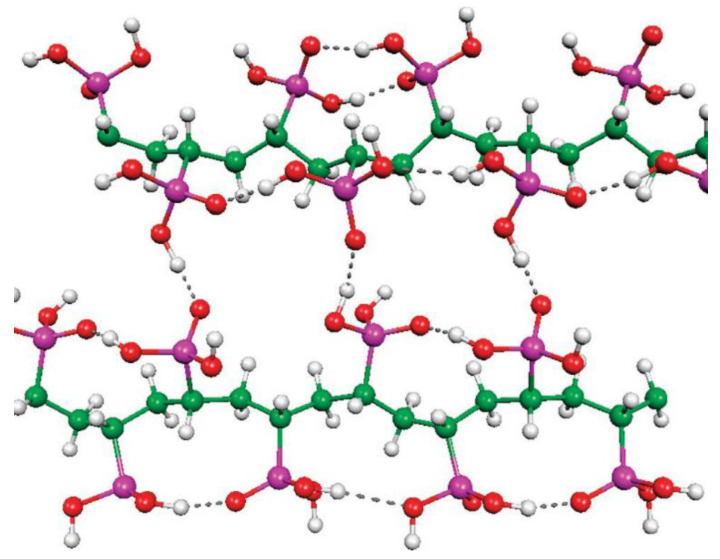
Molecular structure and hydrogen-bonding network of the model system of PVPA as used in the first-principles molecular dynamics simulations and chemical shift calculations. Hydrogen bonds, which are shown as broken lines, are assumed for hydrogen and oxygen pairs with O^…^H distances below 2.3 Å and O–H^…^O angles above 90°. Reprinted with permission from [165]. Copyright (2007) American Chemical Society.

In water-free proton-conducting solid polyelectrolytes, proton transfer can be carried out only by hydrogen bond breaking and forming due to the absence of mobile species other than protons. The mechanism of the complex proton motion in solid PVPA was studied by Spiess and Coll. by solid-state NMR [165,166]. They confirmed the mobility of the P–OH protons but found that the formation of anhydride fragments leads to a decrease in proton conductivity through the decrease in the number of charge carriers and blockage of charge transport through immobilization of charge carriers together with a hindered reorientation of the anhydride groups. The ^1^H chemical shift of the P–OH protons provided evidence for the presence of a hydrogen-bond network (Figure 19), which allows for proton transport via a Grotthus-type mechanism [165]. Combined ^2^H line shape analysis and ab initio MD calculations [166] characterized the dynamical proton motion via the rearrangement of the H-bonding network, which involves both intra- and inter-chain transfers.

The proton conductivity of PVPA was not as high as expected (~0.8 mS∙cm^−1^ at 140 °C in dry conditions [167]), and the activation energy was around 60 kJ∙mol^−1^ [168]. Since poly(ethylene oxide) (PEO) has a low glass transition temperature (210 K) and contains a huge number of oxygen atoms in the backbone which can form hydrogen bonds with phosphonic acid groups, Meyer and Coll. [167] proposed to build a dynamic hydrogen bond network in PVPA/PEO blends. However, even in homogeneous PVPA/PEO blends although the fast mobility of PEO chains, the proton conductivity decreased with increasing PEO content.

In further studies of the same scientific group, the influence of water sorption on the proton conductivity of PVPA was assessed [149]. High-MW almost atactic polymer (*M*_w_ = 62 kDa) was selected for experiments upon annealing and drying; it was demonstrated that H_2_O and self-condensation products coexist in the sample.

**Scheme 42:**
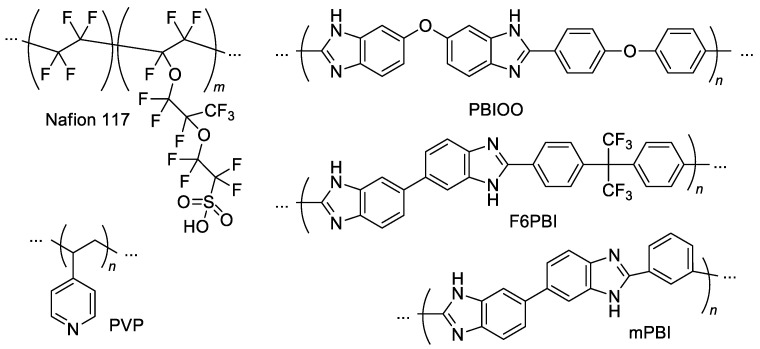
The structures of reference polymer Nafion 117 and polymers used for the preparation of composites.

To improve the characteristics of PVPA-based membranes, Sen and Coll. have studied blends of PVPA and Nafion (Scheme 42), and polymer membranes were prepared by means of film casting from Nafion/PVPA solutions [169]. Nafion/PVPA electrolyte with S/P molar ratio of 1:1 was thermally stable up to 400 °C, the presence of –SO_3_H groups blocked the formation of a phosphonic acid anhydride. The proton conductivity of the Nafion/PVPA blend membrane at 130 °C in an anhydrous state was 1.1 × 10^−5^ S∙cm^−1^. Under humidified conditions, the conductivities of the blends increased by four orders of magnitude, up to 1.2 × 10^−2^ S∙cm^−1^.

The effect of the basic polymeric component in the composite with PVPA was studied by Zhang and Coll. on the example of PVPA-treated SiO_2_/poly(4-vinylpyridine) (PVP, Scheme 42) nanocomposite [170]. PVP-decorated SiO_2_ nanoparticles (diameter of the silica core 7–10 nm), containing 94 wt% of PVP, were treated by aq. PVPA solution in different N/P molar ratios, and freeze-dried. The proton conductivity of the membranes obtained was up to 10 mS∙cm^−1^ under humidified conditions (for 80 wt% PVPA composite) and inferior to PVPA-based membrane.

PVPA/mPBI (Scheme 42) blend membranes were prepared using DMSO polymer solutions and studied by Sharif and Coll. in 2021 [171]. The activation energies of proton transport were calculated in the ranges of 7.1–40.8 kJ∙mol^−1^ and 37.4–49.7 kJ∙mol^−1^ at RH = 100 and 0%, respectively. H_3_PO_4_-doped PVPA/mPBI (1:1 mol/mol) have demonstrated the highest proton conductivity of 79.6 mS∙cm^−1^ at 150 °C, which is comparable to Nafion membrane conductivity (mS∙cm^−1^) under humidified condition at 80 °C. The methanol permeabilities of the blend membranes were about 1000 times lower than that of Nafion.

The complex character of the proton conductivity of PVPA (as well as evident problems arising from the formation of anhydrides at elevated temperatures) prompted the researchers to synthesize and study VPA copolymers. So, for example, free-standing films were obtained from copolymers of VPA and 1-vinyl-1,2,4-triazole (49–67 mol% of VPA) by evaporation of the aqueous solutions and studied [98]. Suddenly, the formation of phosphonic acid anhydride in copolymers was comparable to that in pure PVPA (~30–40% before and ~60% after annealing at 160 °C, ^31^P magic angle spinning (MAS) NMR data). The proton conductivities of the copolymer with maximum VPA content in an anhydrous state was ~1 mS∙cm^−1^ at 120 °C. The hydrogen-bonding network of the copolymers was analyzed and the presence of two different types of hydrogen bonding, O–H^…^O and O–H^…^N, was proved by ^1^H double-quantum MAS NMR. However, at high temperatures (140 °C) highly mobile acidic protons were only detected for O–H^…^O bonding. Copolymers of VPA with 5-(methacrylamido)tetrazole (2:1 comonomer ratio) have demonstrated the maximum proton conductivity of 10 mS∙cm^−1^ at 150 °C [105]. The copolymer of VPA and styrene was studied in mixing with 1-propylimidazole [172], but the results have been mediocre (good thermal stability but very low conductivity even in the presence of water).

PVPA-grafted poly(vinylidene fluoride) (see Section 2.4.5) membranes were prepared from the DMF solutions [140], and the maximum proton conductivity of 2.3 mS∙cm^−1^ was detected at 150 °C for copolymer with 20 wt% PVPA content.

The idea of the use of PVPA/inorganic composites as electrolyte membranes was tested in the study of Aslan and Bozkurt [62] who prepared PVPA/TiO_2_ composites containing 5, 10, 15, or 20 wt% of TiO_2_. The maximum proton conductivity under water-free conditions was detected for 10% TiO_2_ composite at 120 °C and found to be 4 mS∙cm^−1^. For PVPA/BN composites (3, 5, 10, and 15 wt% of the hexagonal BN) high thermal stability was detected (up to 200 °C), and maximum proton conductivity was measured for PVPA-15%HBN as 6.51 mS∙cm^−1^ at 150 °C in anhydrous conditions [61].

#### 3.4.2. Polyelectrolytes Based on Other Non-Fluorinated Polymers

PmPPA (see Scheme 40) [148] has demonstrated high thermal stability (up to 300 °C) without softening before decomposition. Temperature-dependent conductivity measurements under 1 atm water vapor pressure revealed that the conductivity of PmPPA amounted to 6 mS∙cm^−1^ at 115 °C and decreased to 2 mS∙cm^−1^ at a higher temperature. Without humidification, no reproducible results were obtained, which was attributed by the authors to irreversible condensation reactions, so PmPPA can be used as a proton conductor in the presence of water, but not in the dry state.

AIBA-initiated free-radical polymerization of styryl phosphonic acids with –CH_2_O(CH_2_)_n_– (n = 2, 6) spacers between the aromatic ring and P atom (Scheme 8) resulted in the formation of PCPAs with relatively high thermal stability (no anhydride formation up to 140 °C) [49]. The proton conductivities were found to be ~0.26 mS∙cm^−1^ at 140 °C under nominally anhydrous conditions, and the length of the alkanediyl spacer did not affect the conductivity. Functionalized poly(styrene-ethylene/butylene-styrene) block copolymers of an even more simple structure (–CH_2_– spacers between the aromatic ring and –P(O)(OH)_2_, see Section 2.4.3) have demonstrated a lowering of the tensile strength and an increase in proton conductivity. With an increase in the degree of phosphonation [130], the maximum value of protonic conductivity at 140 °C was 5.82 mS∙cm^−1^.

Although fluorinated polymers as polyelectrolytes are discussed in the next section, we felt it more appropriate to consider –CH_2_CF_2_P(O)(OH)_2_-functionalized polystyrene [50] just after non-fluorinated analogs. The IEC of this polymer was 7.3 mmol∙g^−1^, and the conductivity of the dried sample at 120 °C reached 0.018 mS∙cm^−1^.

A similar study with a variation of the length of –(CH_2_)_n_– spacers between ‘PE’ backbone and –P(O)(OH)_2_ groups was conducted by Ueda and Coll. [45]. Phosphonated poly(acrylates) were obtained via free-radical polymerization of CH_2_=CHC(O)O(CH_2_)_n_P(O)(OEt)_2_ (n = 2, 4, 6) followed by hydrolytic cleavage, polymer membranes were prepared by cross-linking with the use of 5 wt% of benzoyl peroxide. The IEC values were 5.44, 4.76, and 4.09 mequiv∙g^−1^, respectively. These membranes have demonstrated high proton conductivity under humidification at 80 °C, and the sample based on acrylate with the longest –(CH_2_)_6_– spacer was not inferior to Nafion 117 (Scheme 42) in conductivity.

In the development of proton-exchange membranes, star polymers may have some advantages compared to linear analogs owing to the compact and branched architecture of star polymers. An original approach to similar polymers was proposed by Müllen and Coll. [74], core-shell macromolecule containing polyaromatic core and polystyrene with –CH_2_P(O)(OH)_2_ groups in the position *para* (Figure 11a) was synthesized and studied. The polymer showed relatively high thermal stability (up to 180 °C), and no anhydride formation was detected by ^31^P MAS NMR. The maximum conductivity of 3.9 mS∙cm^−1^ was detected in a humidified atmosphere at 115 °C.

**Figure 20:**
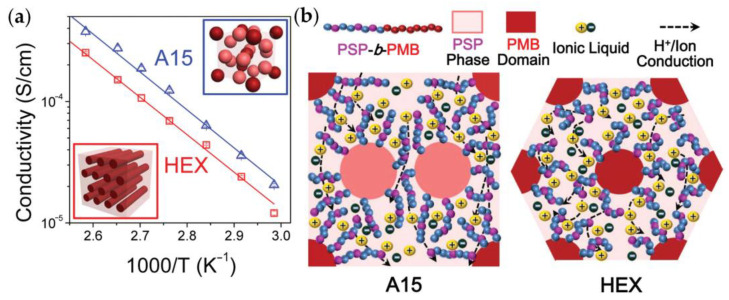
(**a**) Ionic conductivity of [2E4MIm][TFSI]-containing composites of copolymer (11 styrene units with 4 –P(O)(OH)_2_ groups and 18 methylbutylene units) with different thermal histories; (**b**) Schematic drawings that illuminate ion-conduction pathways across PSP phases for A15 and HEX structures. Reprinted with permission from [138]. Copyright (2016) Wiley-VCH GmbH.

Phosphonated poly(styrene-*b*-methylbutylene)s (see Scheme 35) were used in the preparation of membranes, containing ionic liquids (obtained from imidazole (Im), 2-methylimidazole (2MIm) or 2-ethyl-4-methylimidazole (2E4MIm) and bis(trifluoromethane)sulfonimide (TFSI)) [137]. The introduction of alkyl substituents in imidazole resulted in increase of the proton conductivity in the temperature range of 60−150 °C, the maximum value of 2 mS∙cm^−1^ was reached at 150 °C. The studies of the composites with ionic liquids were continued [138] mostly in view of self-assembled morphologies. It was demonstrated that with minimal changes in the numbers of comonomer units in copolymer, and depending on the type of the ionic liquid and thermal history, well-defined lamellae, gyroid, hexagonal cylinder (HEX), body-centered cubic, and A15 lattice morphologies are formed. The samples having the A15 lattice showed higher morphology factors (0.83–0.96) than those with the HEX phases (0.42–0.69), this structural advantage of the A15 lattice over the HEX for constructing less tortuous ion-conduction pathways resulted in higher proton conductivity (Figure 20).

**Figure 21:**
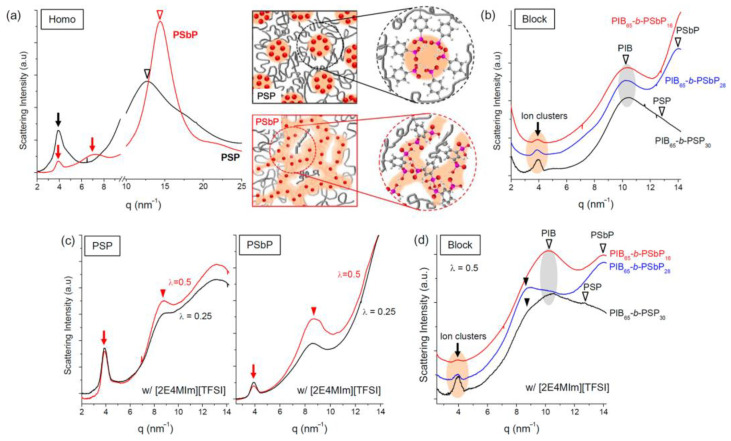
WAXS profiles of phosphonated polymers in the presence and absence of ionic liquids: (**a**) neat homopolymers; (**b**) neat block copolymers; (**c**) [2E4MIm][TFSI]-containing homopolymers; (**d**) [2E4MIm][TFSI]-containing block copolymers. Ion clustering in PSP and PSbP (right panels of (**a**)). λ values in (**c**,**d**) indicate concentrations of ionic liquid. Characteristic peaks are indicated by ↓, ▼, and ▽. Reprinted with permission from [51]. Copyright (2018) American Chemical Society.

Block copolymers of diethyl styryl phosphonate or diethyl styryl bis(phosphonate) (SP and SbP, respectively, see Scheme 9) were synthesized by ATRP, –OC(O)CMe_2_Br-functionalized poly(isobutylene) (pIB) was used as a macroinitiator; homopolymers were also obtained [51]. After hydrolytic cleavage (see Section 2.3.4), poly(phosphonic acid)s were dissolved in THF/MeOH or mixed with Im, 2MIm, or 2E4MIm / TFSI-based ionic liquids in THF/MeOH media, polyelectrolite membranes were obtained by solvent casting and subsequent vacuum drying. Wide-angle X-ray scattering (WAXS) profiles of the membrane samples (pSP, pSbP, pIB_66_-*b*-pSP_30_, pIB_66_-*b*-pSbP_16_, pIB_66_-*b*-pSbP_28_ without and with ionic liquids, Figure 21) and results of the small-angle X-ray scattering (SAXS) studies showed that block copolymers display long-range ordered self-assembled morphologies, in pSbP-based polymers ion aggregation was avoided featuring the confinement of polymer chains to few-nanometer domains.

**Figure 22:**
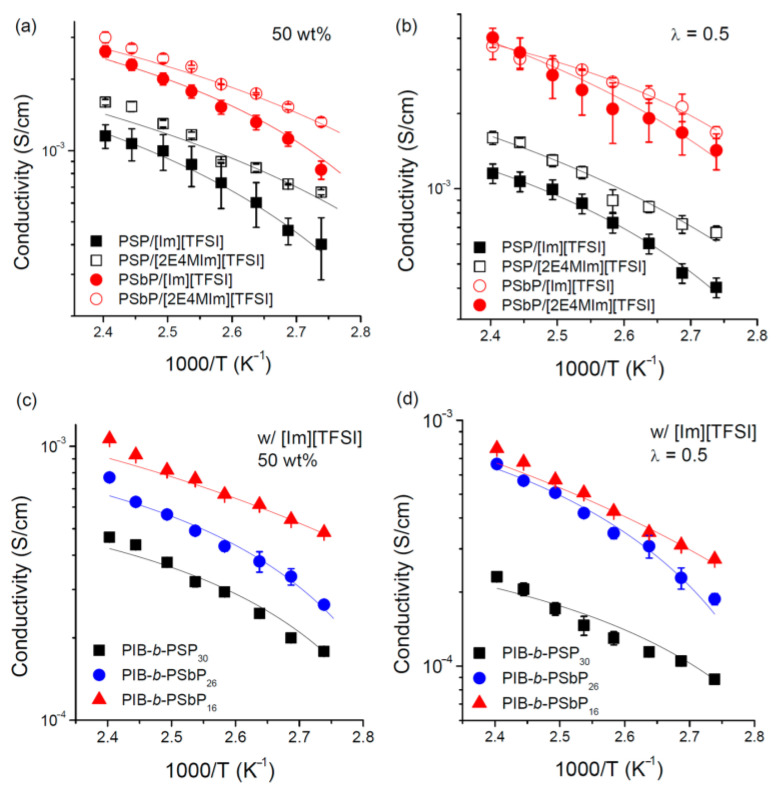
Temperature-dependent through-plane conductivities of (**a**,**b**) ionic liquid-containing phosphonated homopolymers and (**c**,**d**) phosphonated block copolymers. The solid lines in (**a**–**d**) indicate VTF fits. Reprinted with permission from [51]. Copyright (2018) American Chemical Society.

In the presence of ionic liquids, virtually homogeneous ionic phases with suppressed ion clustering were detected for pSbP-based block copolymer. pIB_66_-*b*-pSbP_16_-based membranes have demonstrated the highest conductivity despite lower IEC presumably due to a narrow polymer domain with excluded ion aggregation (Figure 22). Given that the *T*_g_ of pSbP is more than 20 °C higher than the *T*_g_ of pSP, the use of pSbP-based membranes appears a promising way for improving both the conductivity and mechanical strength of solid-state polyelectrolytes.

Polystyrene, containing 1,4-perfluorophenylene linkage between aryl and –P(O)(OH)_2_ fragments (see Scheme 10), under anhydrous conditions at 120 °C exhibited a conductivity of 9.91 × 10^−7^ S∙cm^−1^ [52].

#### 3.4.3. Polyelectrolytes Based on Fluorinated Polymers

For phosphonated copolymers (CF_2_CFClCH_2_CH(OCH_2_CH_2_X))_n_ (X = Cl, H, P(O)(OH)_2_) with different degrees of phosphonation, the dependence of the conductivity on ionic exchange capacity (IEC) was investigated at 20 °C and 95% relative humidity [129]. The level of conductivity was in the range of 0.02–20 mS∙cm^−1^, which is comparable to the Nafion 115 (up to ~50 mS∙cm^−1^). The conductivity was strongly dependent on the IEC: the conductivities of the copolymer with 36% phosphonate functionalization (IEC of 2.9 mequiv∙g^−1^) and 93% phosphonate functionalization (IEC of 7.0 mequiv∙g^−1^) differed by almost 3 orders of magnitude. When decreasing relative humidity from 95 to 25%, the conductivity values decreased tenfold. An increase in the temperature from 90 to 120 °C resulted in an increase of the proton conductivity by twice. This result indicates that water molecules and phosphonic acid hydrogen bonding are involved in the proton conduction mechanism.

**Figure 23:**
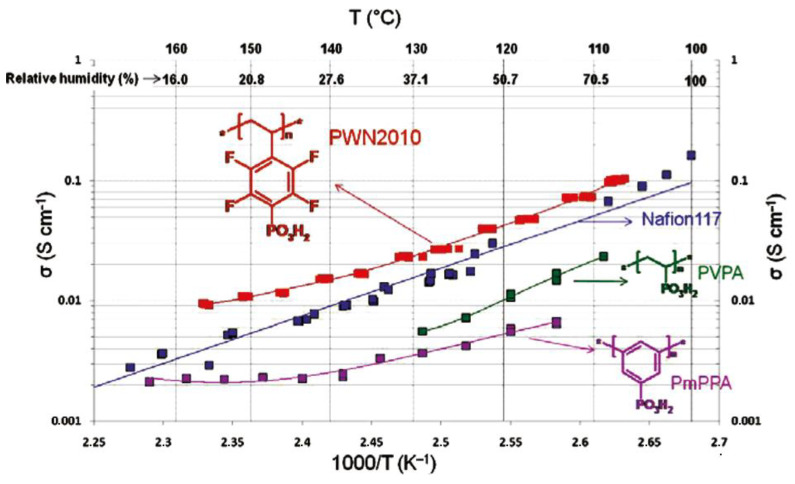
The conductivity of PWN2010 as a function of temperature at water partial pressure of p(H_2_O) = 1 atm. Conductivities of Nafion 117, PVPA, and PmPPA, measured at the same conditions and setup, are given as a comparison. Reprinted with permission from [131]. Copyright (2011) American Chemical Society.

Around the same time, one first and promising step toward the development of efficient solid-state polyelectrolytes has been made by Atanasov and Kerres [131]. Poly(4-vinyl-2,3,5,6-tetrafluorophenylphosphonic acid) (PWN2010) (see Scheme 33) with a degree of phosphonation ~90% and IEC = 7 mmol∙g^−1^ was found to be thermally stable up to 349 °C, and has not *T*_g_. The water uptake of PWN largely exceeded the one of Nafion 117, whereas the numbers of the water molecules per functional group (λ) of PWN2010 and Nafion 117 were similar. The conductivity of PWN2010, measured at 1 atm water vapor pressure (see Figure 23), was found to be by a factor of 4–10 higher than similar phosphonated polymers such as PVPA and PmPPA. It was even higher than the conductivity of Nafion 117 (Scheme 42), being up to two times larger at T = 155 °C. Similar to PmPPA, PWN2010 showed reduced dependence on the conductivity from the water content at higher temperatures (T = 135–155 °C). The absence of conductivity hysteresis indicated the absence of the anhydride formation in stark contrast to PVPA and PmPPA.

In [133], Atanasov and Coll. reported the results of further studies of PWN. They convincingly showed that PWN really does not form anhydride at moderately elevated temperatures. Even at 250 °C 48% dehydration required annealing within 5 h! At 150 °C in dry N_2,_ the proton conductivity was 0.4 mS∙cm^−1^. Stable polymer films were prepared by blending PWN with poly(benzimidazole) PBIOO (Scheme 42), which after doping with PA reached a peak power density of about 230 mW∙cm^−2^ at 150 °C. Another important aspect in the potential use of PWN is its poor mechanical properties at high degrees of the phosphonation, and in [132] the synthesis and studies of poly(pentafluorostyrene)s with controlled phosphonation degrees (17–66%) were presented. Ion conductivity was found to increase with both temperature and phosphonation degree reaching 57 mS∙cm^−1^ at 120 °C (RH ~90%). In the fuel cell, the blend showed the best peak power density of 400 mW∙cm^−2^ against 350 mW∙cm^−2^ for Nafion 212. A blend membrane consisting of 82 wt% PWN and 18 wt% benzimidazole copolymer F6PBI (Scheme 42) with IEC ~ 2.1 mmol∙g^−1^ was produced and showed better thermal and radical resistance, lower water uptake but similar ion-conductivity. With the use of these blends, full-featured prototypes of the fuel cells were developed and tested.

**Figure 24:**
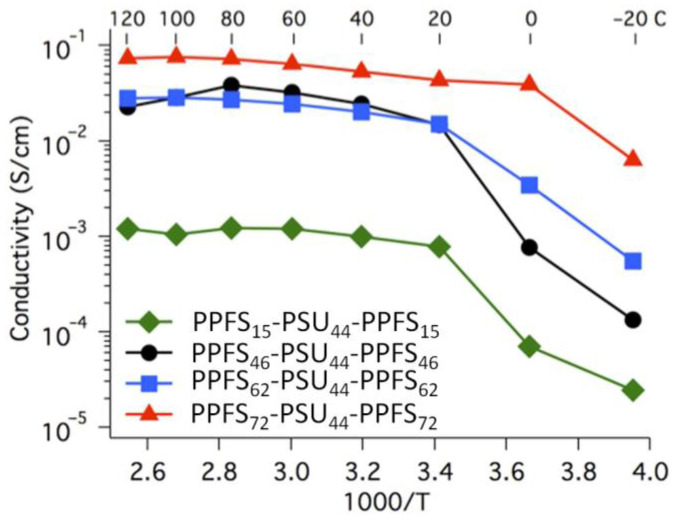
Proton conductivity of block copolymer membranes as a function of temperature under fully hydrated conditions. Reprinted with permission from [136]. Copyright (2013) Wiley-VCH GmbH.

In trying to improve PWN characteristics, triblock copolymers with poly(diaryl sulfone) were synthesized (see Scheme 34) and studied [136]. Copolymer membranes were prepared from N,N-dimethylacetamide solutions. Under hydrated conditions, copolymers have demonstrated high values of proton conductivity (Figure 24).

The conductivity value for copolymer membrane, based on perfluoro-1,1′-biphenyl and diphosphonated hydroquinone (see Scheme 39), reached 92 mS∙cm^−1^ when fully hydrated at ambient temperature [147] which is comparable with standard Nafion membranes [173] and substantially higher than reported for certain other phosphonated polymers [44,129,139].

In [134], the most promising results of the studies of PWN-based polymer membranes were discussed and summarized, and the advantages (film processing, proton conductivity, acid retention with water) and disadvantages (hydrophobicity, thermal stability) of these polymers in comparison with Nafion were demonstrated.

The high efficiency of the synergy of PWN-type polymer and Nafion was demonstrated in the very recent work of Kin and Coll. [135]. They proposed that the presence of highly acidic –SO_3_H groups could enhance the anhydrous proton conduction of fuel cell electrodes due to proton transfer to the phosphonic acid groups. The fuel cell, exhibiting a rated power density of 780 mW∙cm^−2^ at 160 °C, with minimal degradation during 2500 h of operation and 700 thermal cycles from 40 to 160 °C under load, was developed by the results of the study.

### 3.5. Other Applications of Sidechain PCPAs

#### 3.5.1. Sidechain PCPAs as Flame Retardants

The phosphorus-containing FRs surpass halogen-based FRs in safety and are being intensively researched [12]. In a recent review of Özer and Gaan, devoted to phosphorus-based flame retardant coatings for textiles [174], low-MW compounds have mainly been described.

As with main-chain PCPAs, sidechain polyacids have been little studied as FRs. In 2013 Fang and Coll. reported on a study of the construction of Zn^2+^- and Cu^2+^-doped FR coatings on the surface of ramie fabrics [175]. PVPA and branched polyethylenimine/M^2+^ were used in the layer-by-layer assembly technique. As a result, the thermal and fire stability of the coated fabric was improved, e.g., a significant residue (up to 25.2 wt %) was left (<1% for the control). The results of this study were somewhat expanded and corrected one year later [176].

**Figure 25:**
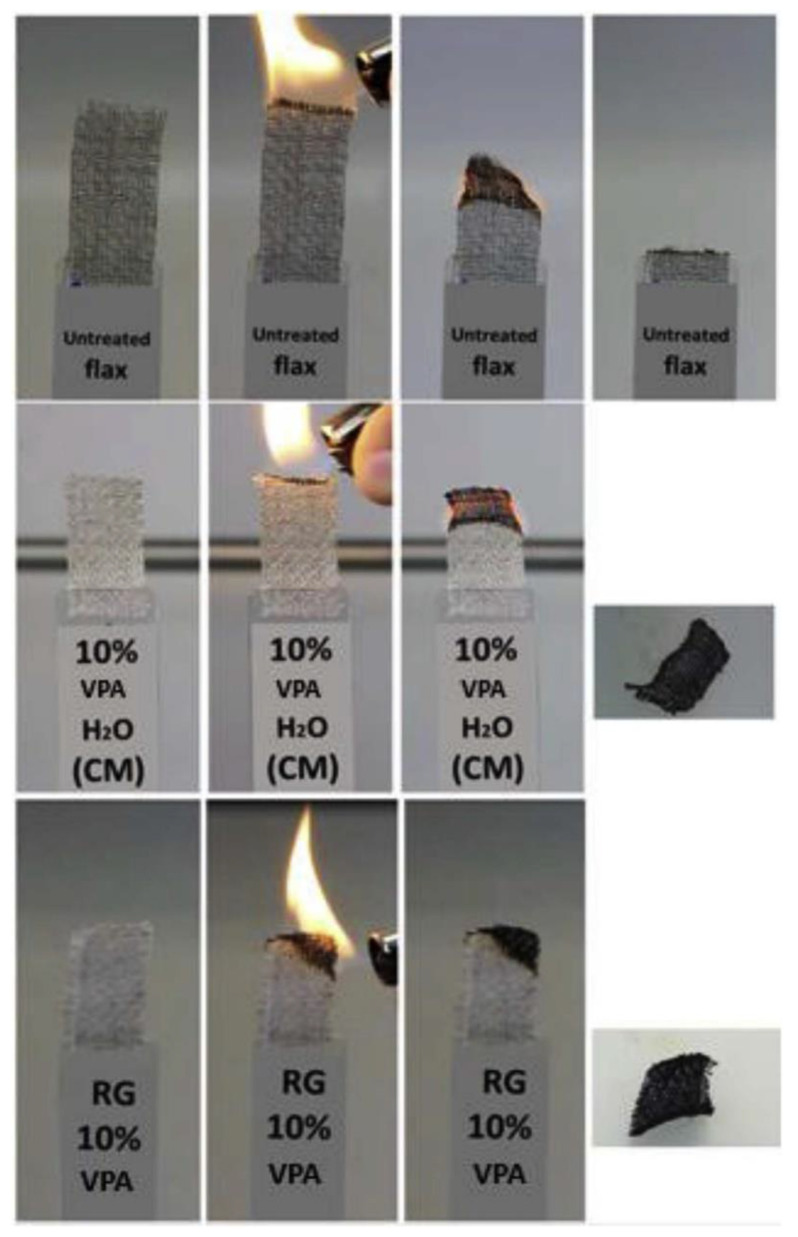
Preliminary fire test on untreated (top), chemically modified (middle), and irradiated (bottom) flax fabrics. Reprinted with permission from [141]. Copyright (2018) Elsevier B. V.

El Hage and Coll. had chosen not to use metal salts, and studied different methods of VPA grafting [141] (Figure 7). Flame retardant properties of VPA-grafted flax fabrics can be explicitly illustrated by Figure 25, thus confirming the correctness of a chosen way.

**Scheme 43:**
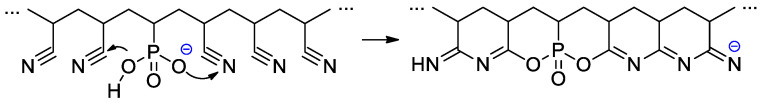
The proposed mechanism, and the effect of phosphonate and phosphonic acid on cyclization during heat treatment of acrylonitrile copolymers. [103].

As was shown in [103], even small amounts of VPA in copolymer with acrylonitrile or acrylonitrile/methyl methacrylate reduces significantly the flammability of the materials. The authors assumed that the presence of –P(O)(OH)_2_ groups in acrylonitrile-based polymers accelerates intramolecular cyclization of the nitrile units (Scheme 43).

Copolymers of methyl methacrylate and the most synthetically available phosphate-containing acrylate-type monomer MEPN (see Scheme 7) were studied by Marić and Coll. in 2020 [109]. They showed that the char yield content increased from 1.2 to 24.4 wt% for copolymers containing 6 to 28 mol% of MEPN. The limiting oxygen index (*LOI*) value (the minimum fraction of oxygen in an O_2_/N_2_ mixture supporting the burning of a vertical sample) was estimated by Van Krevelen empirical Equation (3) for halogen-free polymers [177]:

*LOI* = 17.5 + 0.4 × *CY* (3)

where *CY* is char yield. The *LOI* of pure poly(methyl methacrylate) and copolymer with 28 mol% MEPN content were 17.8 and 27.3 respectively. Note that the *LOI* of pure poly(MEPN) was 34.1.

#### 3.5.2. Sidechain PCPA-Based Polymer Networks for Enzyme Immobilization

**Scheme 44:**
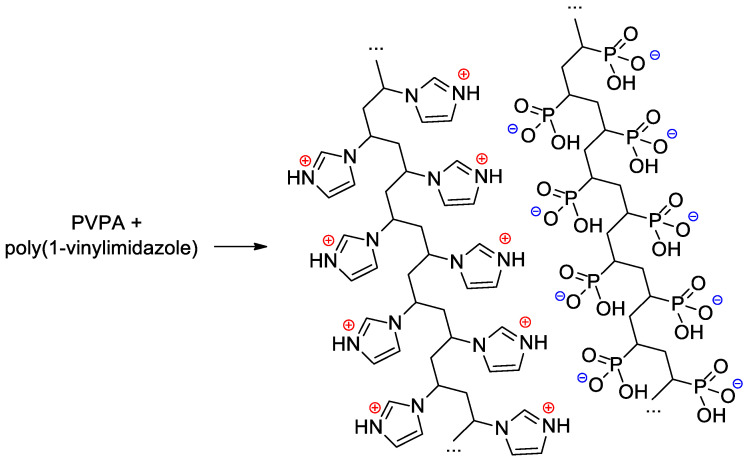
Protonation of PVPA and poly(1-vinylimidazole) with a formation of polyelectrolyte network [178].

In the study of Toppare and Coll. [178], proton conducting polymer blend was prepared by mixing PVPA with poly(1-vinylimidazole) at various ratios (Scheme 44). The polymer network having the most suitable stoichiometric ratio for substantial proton conductivity was used for the immobilization of invertase. Despite the fact that the value of the Michaelis-Menten constant (*K*_M_) for immobilized invertase was 3.5 times lower than the *K*_M_ of the free enzyme (6.8 vs. 24.3 mM), the use of polymer matrix allowed to have the activity of the enzyme kept constant after 25 repetitive usages.

## 4. Conclusions

In our review, we tried to show all the diversity of the synthetic approaches to sidechain PCPAs and the great potential of their applications. Sidechain PCPAs are different from other organic polyacids by higher acidity and, more significantly, by the ability to participate in biomineralization processes. The high chemical stability of the backbone of the sidechain PCPAs is a clear advantage in the development of the solid-state polyelectrolytes but is a serious obstacle to their use in tissue engineering and bone surgery.

Both for main-chain and sidechain PCPAs, the potential of the current polymer chemistry and science of catalysis remains largely unexploited. The very idea to combine polyester (or other) biodegradable backbone with –P(O)(OH)_2_-functionalized substituents has not yet been implemented despite its apparent promise.

## Figures and Tables

**Table 1:** Characteristics of VPA/CH_2_=CHP(O)(OMe)_2_ copolymers obtained by SEC-MALLS measurements (sample concentration of 1 mg/mL) [73].

Polymer	*M*_n_, kDa	*M*_w_, kDa	*Ð*_M_	*R*_h_(Q), nm ^1^	Dt, cm^2^∙s^−1 2^	Intrinsic viscosity, mL∙g^−1^	MHS ^3^
PVPA	27.75	34.20	1.23	3.50	7.40 × 10^−7^	36.36	0.7197
VPA:DMVP 1:1 ^4^	8.78	12.70	1.45	2.48	1.22 × 10^−6^	16.03	0.6432
VPA:DMVP 2:1	11.90	15.13	1.27	3.03	1.10 × 10^−6^	19.05	0.6331
VPA:DMVP 3:1	11.33	17.10	1.51	2.93	1.38 × 10^−6^	20.37	0.6878
VPA:DMVP 4:1	16.12	19.12	1.19	5.32	1.47 × 10^−6^	22.28	0.6794

^1^ *R*_h_—hydrodynamic radius. ^2^ Dt—diffusion coefficient. ^3^ MHS—the exponent from Mark-Howink-Sakurada plots. ^4^ DMVP—dimethyl vinyl phosphonate, (MeO)_2_P(O)CH=CH_2_.

## Data Availability

Not applicable.
